# Resin Infiltration for the Management of Hypomature Amelogenesis Imperfecta: A Case Report

**DOI:** 10.1155/crid/7795936

**Published:** 2026-09-02

**Authors:** Elsa Garot, Julia Estivals, Ana Ribeiro, Anais Cavaré, Olivia Kérourédan, Julien Van Gils

**Affiliations:** ^1^ UFR des Sciences Odontologiques, Univ. of Bordeaux, Bordeaux, France; ^2^ PACEA UMR 5199, Univ. of Bordeaux, Pessac, France; ^3^ Centre de Compétence des Maladies Rares Orales et Dentaires, CCMR O-Rares, University Hospital of Bordeaux, Bordeaux, France; ^4^ C.H.U. of Bordeaux, Bordeaux, France; ^5^ Centre de Compétence des Maladies Osseuses Constitutionnelles, CCMR MOC, University Hospital of Bordeaux, Bordeaux, France; ^6^ Centre de Compétence des Maladies Rares du Métabolisme du Calcium et du Phosphate, CCMR CaP, University Hospital of Bordeaux, Bordeaux, France; ^7^ Univ. of Bordeaux, BioTis UMR 1026, INSERM, Bordeaux, France, inserm.fr; ^8^ Department of Medical Genetics, University Hospital of Bordeaux, Bordeaux, France; ^9^ Univ. of Bordeaux, INSERM U1211, Bordeaux, France

**Keywords:** adolescent, case report, esthetic dentistry, hypomature enamel, minimally invasive dentistry

## Abstract

**Objective:**

We are aimed at describing the clinical and psychosocial benefits of resin infiltration as a minimally invasive treatment option for hypomature amelogenesis imperfecta in a child. Hypomature amelogenesis imperfecta is a rare hereditary enamel defect that significantly compromises dental esthetics, function, and psychosocial well‐being. Conventional management often involves invasive restorative approaches such as composites, veneers, or crowns, which may require removal of sound tooth tissue and pose challenges in pediatric patients.

**Clinical Considerations:**

An 11‐year‐old girl presenting with hypomature amelogenesis imperfecta and severe esthetic concerns associated with impaired oral health–related quality of life was treated using an erosion infiltration technique with resin (Icon, DMG). Following enamel surface conditioning, a low‐viscosity resin infiltrant was applied to penetrate and mask hypomature enamel opacities. The procedure led to immediate and significant esthetic improvement, with enhanced enamel translucency and high patient satisfaction. Esthetic outcomes remained stable over a 3‐year follow‐up period, including during orthodontic treatment, and were associated with a marked improvement in oral health–related quality of life.

**Conclusions:**

This case highlights the potential of resin infiltration to offer a conservative alternative to conventional restorative methods in hypomature amelogenesis imperfecta, with meaningful improvement in dental esthetics and oral health–related quality of life. Nevertheless, further clinical studies and long‐term follow‐up are required to validate its durability and to establish standardized treatment protocols.

**Clinical Significance**

This case demonstrates that resin infiltration can be a valuable minimally invasive approach for the esthetic management of hypomature amelogenesis imperfecta (AI). By preserving enamel tissue and avoiding invasive restorative options, it offers an effective solution to improve dental appearance and patient self‐esteem at an early age. Its relevance to esthetic dentistry lies in providing a conservative, painless, and well‐accepted technique that enhances enamel translucency and harmonizes tooth color while respecting the principle of tissue preservation.

## 1. Introduction

AI is a rare genetic disorder that primarily affects the structure and clinical appearance of the enamel of all or nearly all teeth, both primary and/or permanent [1]. In 1988, Witkop [2] proposed a revised classification of AI based on both the clinical characteristics of enamel defects and the mode of inheritance, distinguishing four main types: Type I (hypoplastic), Type II (hypomaturation), Type III (hypocalcified), and Type IV (hypomaturation–hypoplastic with taurodontisme). This classification was subsequently reconsidered by several authors, as discussed by Crawford et al. [3], who advocated for the incorporation of molecular and genetic data into the diagnostic framework. To date, more than 70 genes have been identified in association with both isolated and syndromic forms of AI [4]. This genetic disorder can occur sporadically or be inherited through various modes of transmission: autosomal dominant, autosomal recessive, or X‐linked [3]. AI may occur as an isolated condition or be associated with other symptoms as part of rare syndromes [3]. A French network reported the genetic findings of 115 individuals diagnosed with AI, who were referred to 16 specialized centers for rare oral and dental diseases across France [4]. Among the 151 sequenced variants, 47 are newly identified and classified as Class 4 or 5. The most commonly detected genotypes in isolated AI were linked to the MMP20 and FAM83H genes. For syndromic AI, the most frequently identified genes were FAM20A and LTBP3 [4]. Several forms of the condition have been identified: hypoplastic, hypomature and hypomineralized. Enamel defects can be qualitative, involving changes in color and/or surface texture or quantitative, involving shape. Studies of various populations have shown prevalence rates of AI ranging from 1:700 to 1:14,000 [3, 5]. Clinically, AI can lead to complications such as rapid tooth wear, esthetic concerns, and dentofacial discrepancies. Evidence from a systematic review indicates that AI significantly impairs oral health–related quality of life (OHRQoL), especially in domains related to esthetics, hypersensitivity, function, and psychosocial well‐being, emphasizing the importance of timely and appropriate dental management [6]. Among 60 children and adolescents (5–17 years) with AI, pain or sensitivity was frequently reported, with 72% experiencing these symptoms often or sometimes. Furthermore, 76% reported being often or sometimes dissatisfied with the appearance of their teeth [7]. This rare disease affects the quality of life of both patients and their parents, not only due to the direct impact on daily functioning, but also because of the limited awareness and absence of standardized treatment guidelines within the healthcare system [8]. As such, AI represents a significant challenge in terms of clinical management and prevention of dental complications. It often requires a multidisciplinary approach involving dentists, orthodontists, and geneticists.

Hypomature AI is a qualitative enamel defect, accounting for 27%–40% of all cases of AI [8]. Although enamel thickness is normal, it is softer and tends to fracture more easily, making it more susceptible to wear. Clinically, the enamel has slightly reduced hardness, lacks translucency, and displays a mottled, opaque appearance with shades ranging from chalky white to brown—often referred to as “snow‐capped teeth” [4]. In hypomature forms, the enamel is less mineralized and shows little radiographic contrast compared with dentin, with similar radiodensities and an abnormal enamel radiopacity reflecting a low mineral density [3, 9]. Hypomineralized and hypomature AI result from a lower mineral content of the enamel and an abnormal formation of its structural organization, leading to a hardness that is significantly lower than that of normal enamel. Furthermore, the decrease in mineral proportion is associated with an increase in protein content. The average mineral content of enamel is reduced in all teeth affected by hypomature and hypocalcified AI, whereas in hypoplastic forms the enamel mineral content ranges from normal to reduced [3, 9].

Hypomature AI is caused by incomplete removal of extracellular proteins during the maturation phase. Enamel maturation begins once the full enamel thickness is achieved and involves the proteolytic breakdown and removal of matrix proteins, along with the growth of hydroxyapatite crystals [10]. The resulting enamel may appear less translucent, more opaque (resembling a snow‐capped appearance), or pigmented. Among the four recognized forms of hypomature AI, the pigmented and snow‐capped variants follow an autosomal dominant inheritance pattern (Types IIA and IIC), whereas two others are X‐linked (Types IIB and IID) [11]. Hypomaturation defects are most likely attributable to pathological effects of the truncated ENAM protein containing a unique 24–amino acid frameshifted C‐terminus [12]. The persistence and accumulation of the p.Asn197Glufs ^∗^25 protein in ameloblasts after the postsecretory transition could impair enamel maturation, giving rise to the hypomaturation phenotype [12].

The most frequent differential diagnosis is dental fluorosis, due to its wide range of presentations—from mild white flecks on the enamel to intense white opacities with irregular, disfiguring stains and hypoplastic areas. Careful clinical history is essential to distinguish it from hypomature AI. Fluorosis may exhibit horizontal white bands corresponding to periods of elevated fluoride exposure, and in some cases, premolars or second permanent molars may be unaffected, reflecting a chronological pattern. In such instances, the patient′s history often reveals excessive fluoride intake, either through habits like swallowing toothpaste during childhood or due to high fluoride levels in the local water supply [3].

The esthetic management of hypomature AI remains particularly challenging, especially in children and adolescents, for whom conventional restorative approaches may be excessively invasive and difficult to justify at an early age. Current therapeutic options are largely limited to composite restorations, veneers, or full‐coverage crowns, all of which involve irreversible enamel removal. Resin infiltration, initially developed for the management of early carious lesions and subsequently extended to hypomineralized enamel defects such as molar‐incisor hypomineralization and fluorosis, has not yet been established as a treatment option for hypomature AI. AI is characterized by increased enamel porosity, retention of the organic matrix, and altered optical properties, all of which contribute to its characteristic clinical appearance. It also explains that these structural alterations may facilitate the penetration of a low‐viscosity infiltrant resin into the porous enamel, thereby modifying its refractive index and reducing light scattering. Consequently, the potential of resin infiltration to improve the esthetic appearance of affected teeth while preserving tooth structure through a minimally invasive approach may be of interest. This clinical report describes the use of a minimally invasive erosion‐infiltration protocol with low‐viscosity resin (Icon, DMG) to manage generalized hypomature AI in an 11‐year‐old patient, with the aim of improving dental esthetics while preserving enamel tissue and respecting the principle of therapeutic gradation.

## 2. Case Presentation

**Figure 1:**
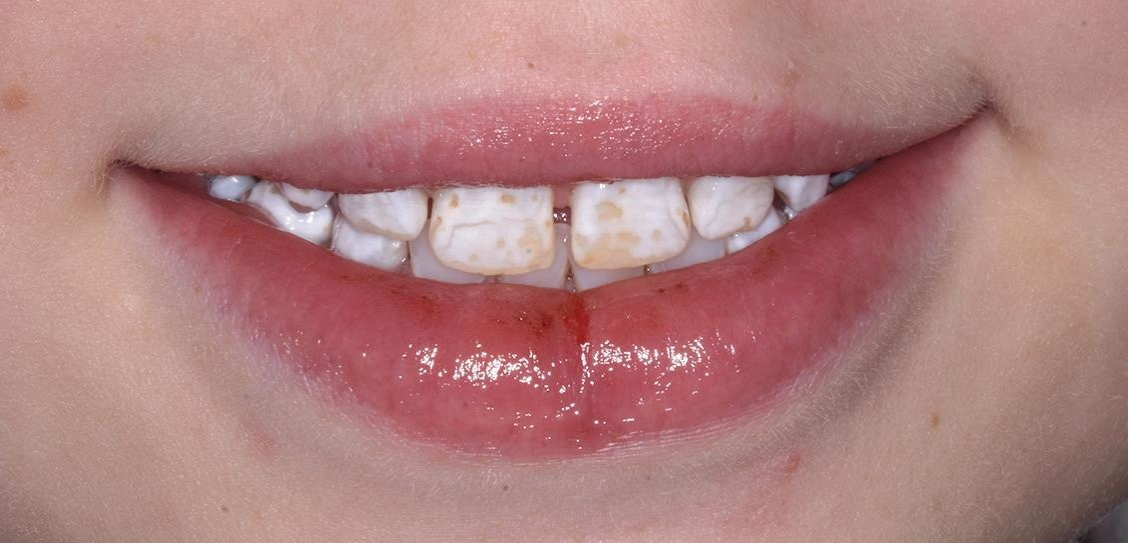
Preoperative extraoral view showing the patient′s smile.

An 11‐year‐old girl presented to the Department of Oral Medicine (CHU de Bordeaux, France) with esthetic concerns affecting all of her upper and lower teeth (Figure 1). She reported being teased by classmates, which has significantly impacted her self‐esteem and caused her to hide her teeth when smiling. The OHRQoL was assessed using the COHIP (Child Oral Health Impact Profile), a child‐adapted version of the OHIP (Oral Health Impact Profile) for children aged 7–17 years. The original version comprises 34 items divided into five subscales: oral health, functional well‐being, social and emotional well‐being, school environment, and self‐image [13]. A short‐form version (COHIP‐SF) was later developed, consisting of 19 selected items grouped into three subscales: oral health, functional well‐being, and a combined subscale covering social/emotional well‐being, school environment, and self‐image. This shortened version can be completed either by the child or the parent, and has demonstrated comparable reliability and validity to the full version [14, 15]. The COHIP‐SF score completed by the patient was 28, indicating a severe impairment of OHRQoL, with substantial functional limitations, emotional distress, and/or social difficulties.

**Figure 2:**
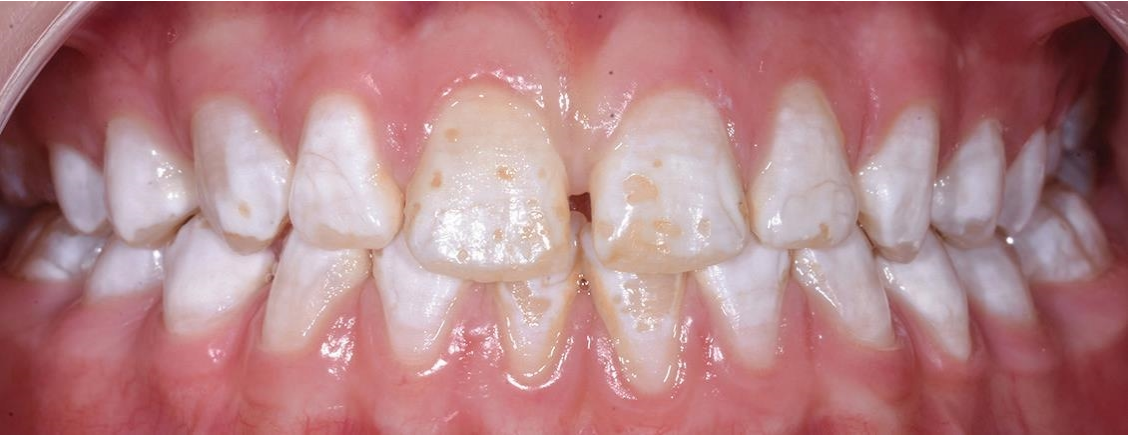
Preoperative intraoral view of the patient with hypomature AI, showing generalized “snow‐capped” white opacities affecting all teeth.

**Figure 3:**
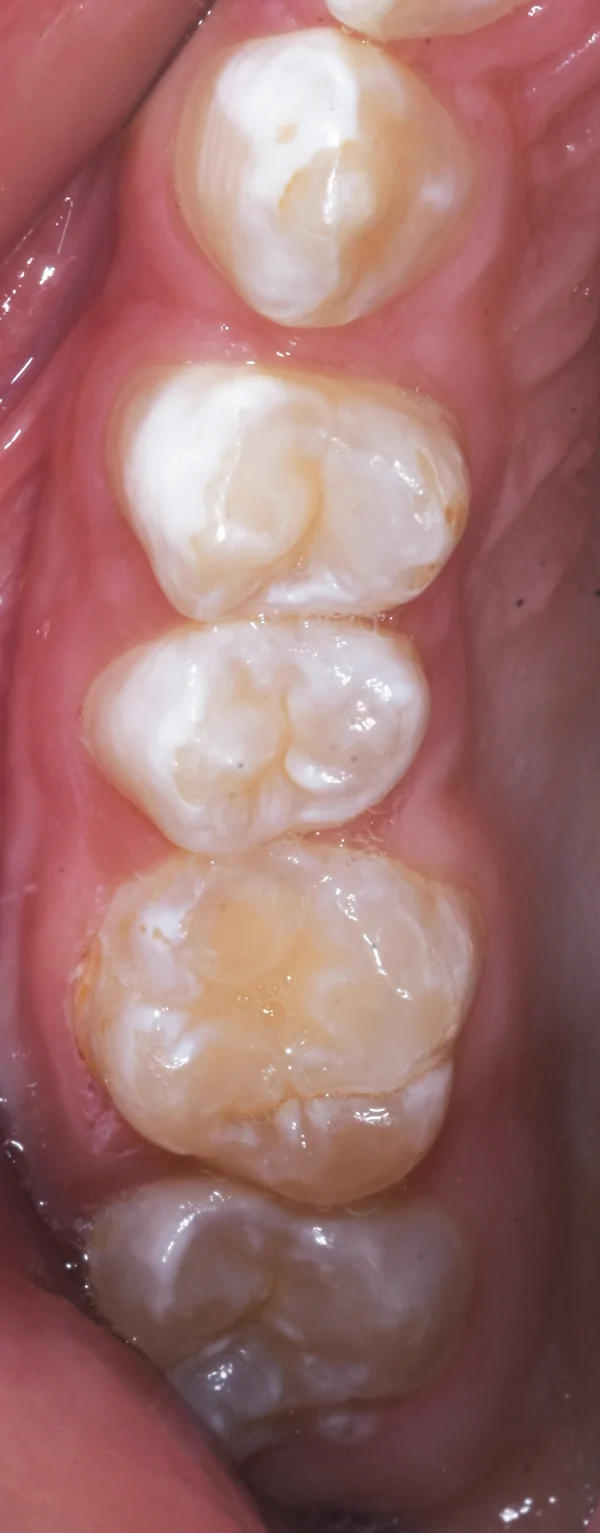
Preoperative occlusal view of the maxillary right quadrant (Quadrant 1).

**Figure 4:**
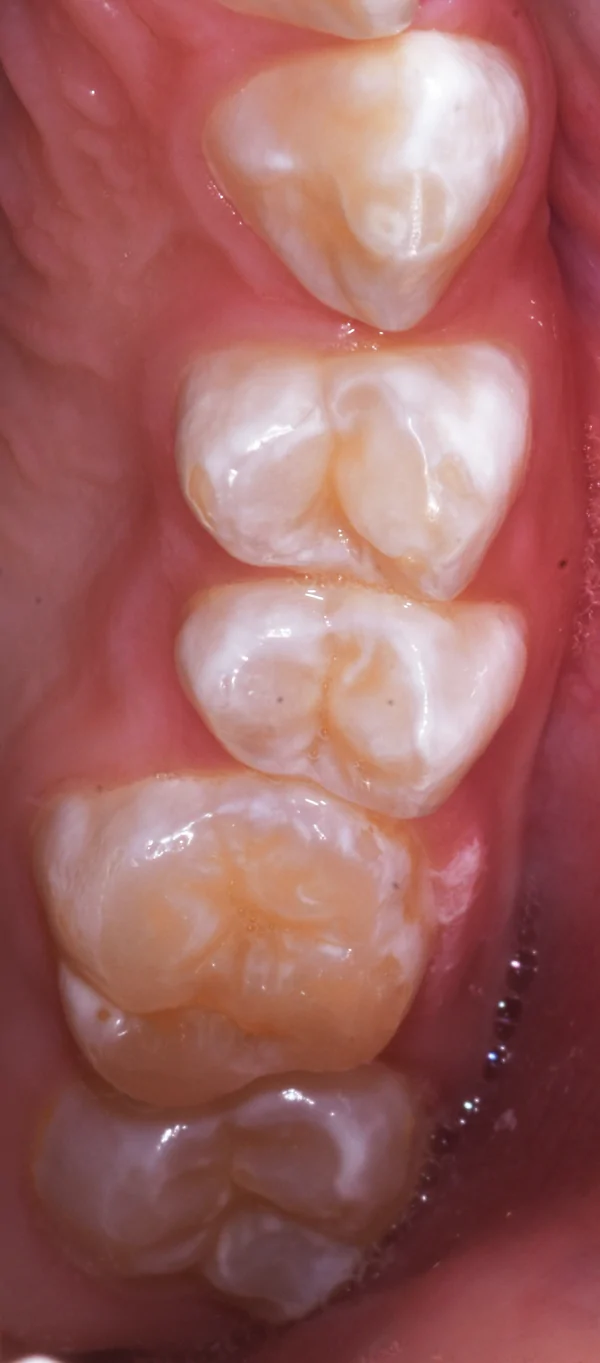
Preoperative occlusal view of the maxillary left quadrant (Quadrant 2).

**Figure 5:**
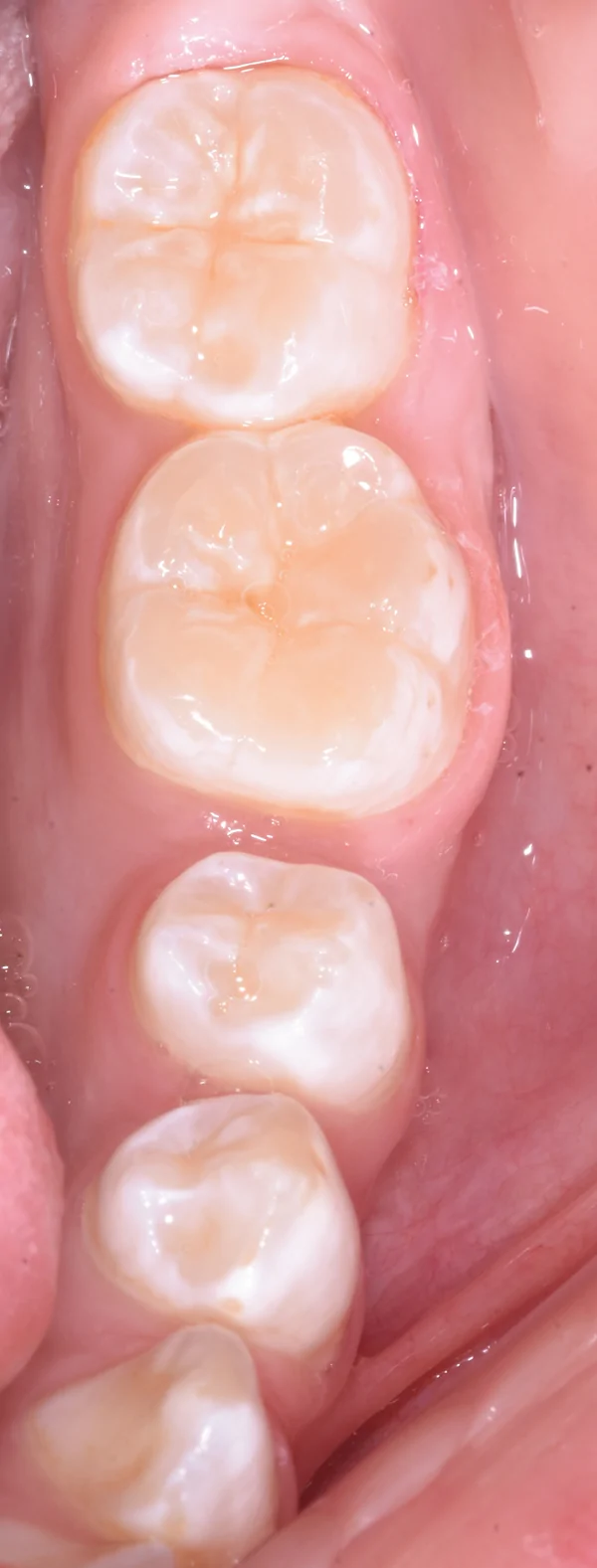
Preoperative occlusal view of the mandibular left quadrant (Quadrant 3).

**Figure 6:**
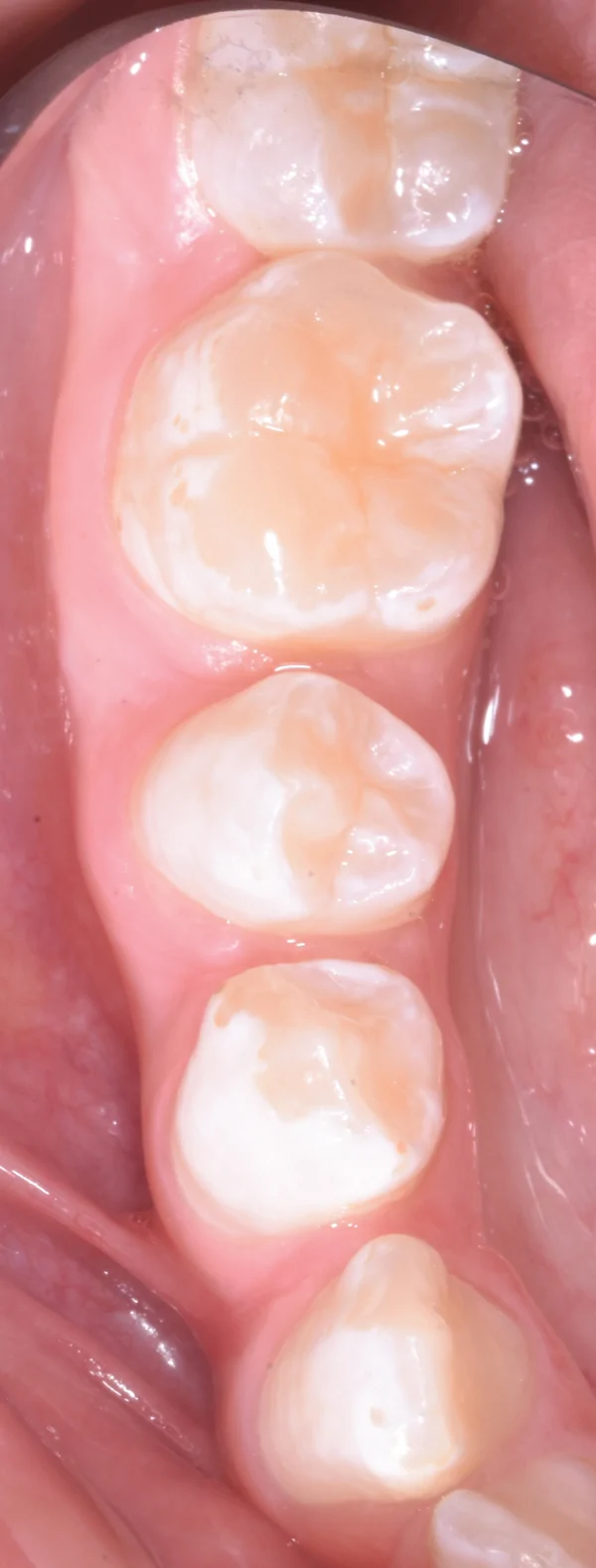
Preoperative occlusal view of the mandibular right quadrant (Quadrant 4).

**Figure 7:**
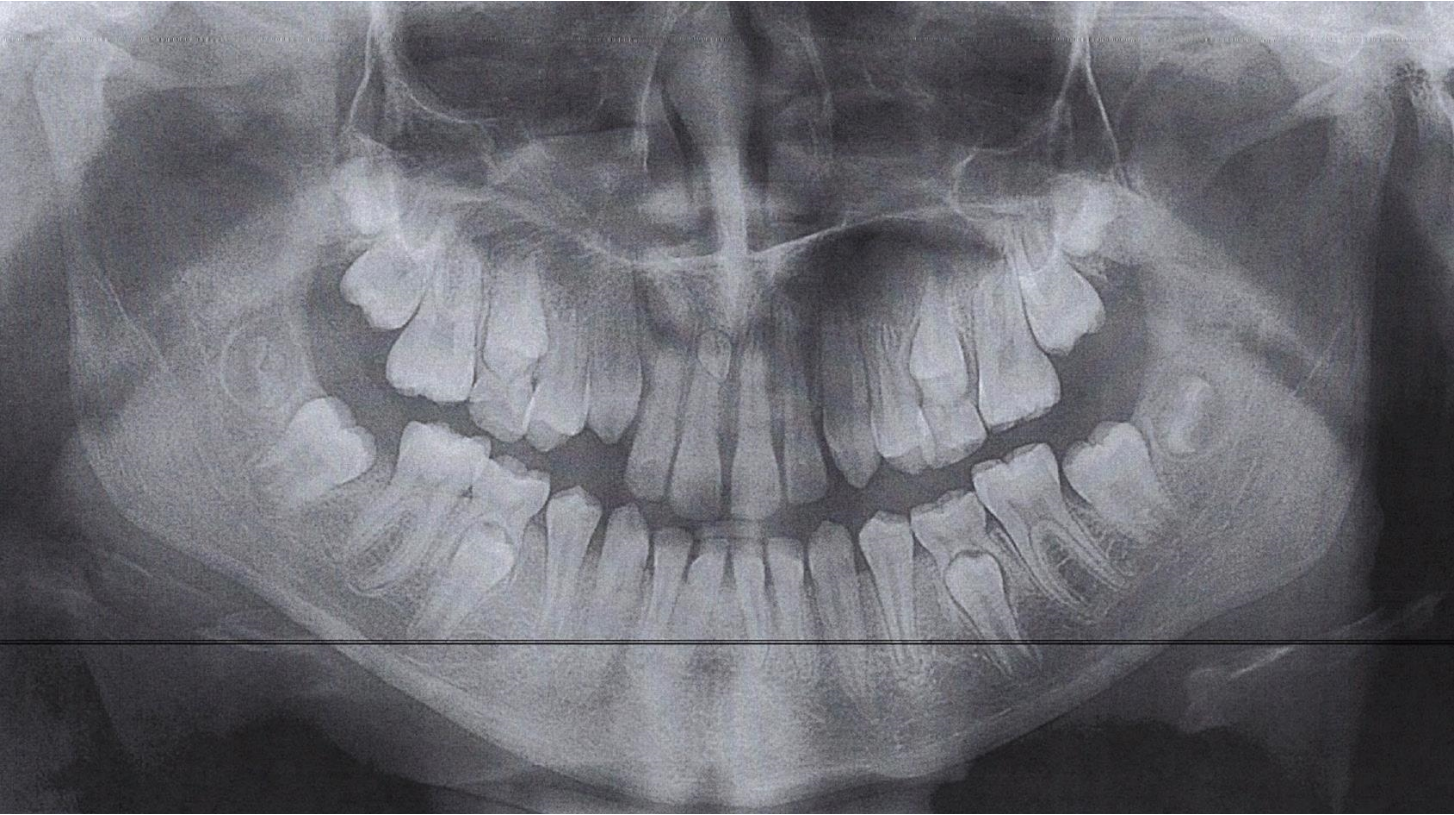
Panoramic radiograph showing a mineral density of the enamel similar to that of dentin, particularly in the incisor and canine regions.

The clinical examination confirmed the diagnosis of AI with generalized enamel structural anomalies affecting all permanent maxillary and mandibular teeth. Significant areas of white opacities are observed in both the anterior and posterior sectors (Figures 2, 3, 4, 5, and 6), associated with minor hypoplasias (Figure 2). The teeth typically present with enamel of normal thickness but it appears opaque with a mottled white, cream, yellow, or brown discoloration and exhibit a snow‐capped appearance on incisal and occlusal surfaces (Figures 2, 3, 4, 5, and 6). The enamel surface is smooth but may be prone to chipping and wear after eruption on first permanent molars (Figures 3, 4, 5, and 6). Affected teeth show reduced translucency, staining, and compromised esthetics (Figure 2). A similar mineral density between enamel and dentin can also be observed on incisors and canines (Figure 7).

**Figure 8:**
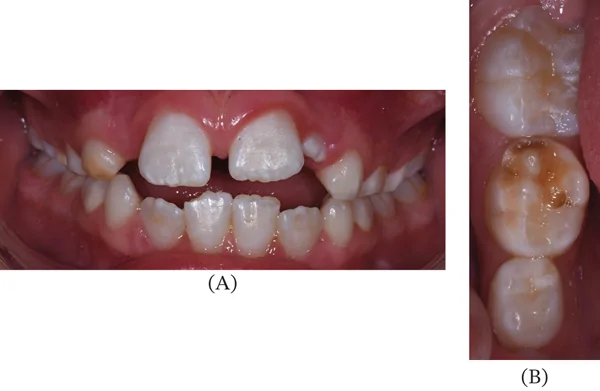
(A) Intraoral view of the patient′s younger sister showing generalized enamel defects consistent with hypomature amelogenesis imperfecta. White opacities affected the permanent maxillary and mandibular incisors, whereas a brown opacity was observed on right primary canine and cream opacity on left primary canine. (B) Occlusal view showing white opacities affecting the mandibular right first permanent molar and the first primary molar, whereas brown opacities were observed on the left second primary molar.

The differential diagnosis was guided by the clinical phenotype, medical history, and the topographic distribution of the structural anomalies. In this case, the differential diagnosis lied between a hypomature form of AI and fluorosis. The fluoride assessment conducted in the patient reveals no history of multiple sources of systemic fluoride exposure during childhood, with the only fluoride use being toothpaste, which would lean the diagnosis toward a hereditary hypomature AI. The distribution of the enamel defects did not follow the chronology of tooth mineralization. Moreover, the primary teeth preserved by the parents exhibited the same characteristics with white spots on her primary molars and incisors. This form of AI also affected her younger sister with white, cream, and brown spots on primary and permanent teeth (Figure 8A,B). Both patients reported no tooth sensitivity. Genetic testing is not routinely performed, and the decision to pursue genetic analysis is made on an individual basis by the patient and their family. As no additional clinical findings suggestive of a syndromic condition were identified, genetic testing was not deemed necessary in the present case.

**Figure 9:**
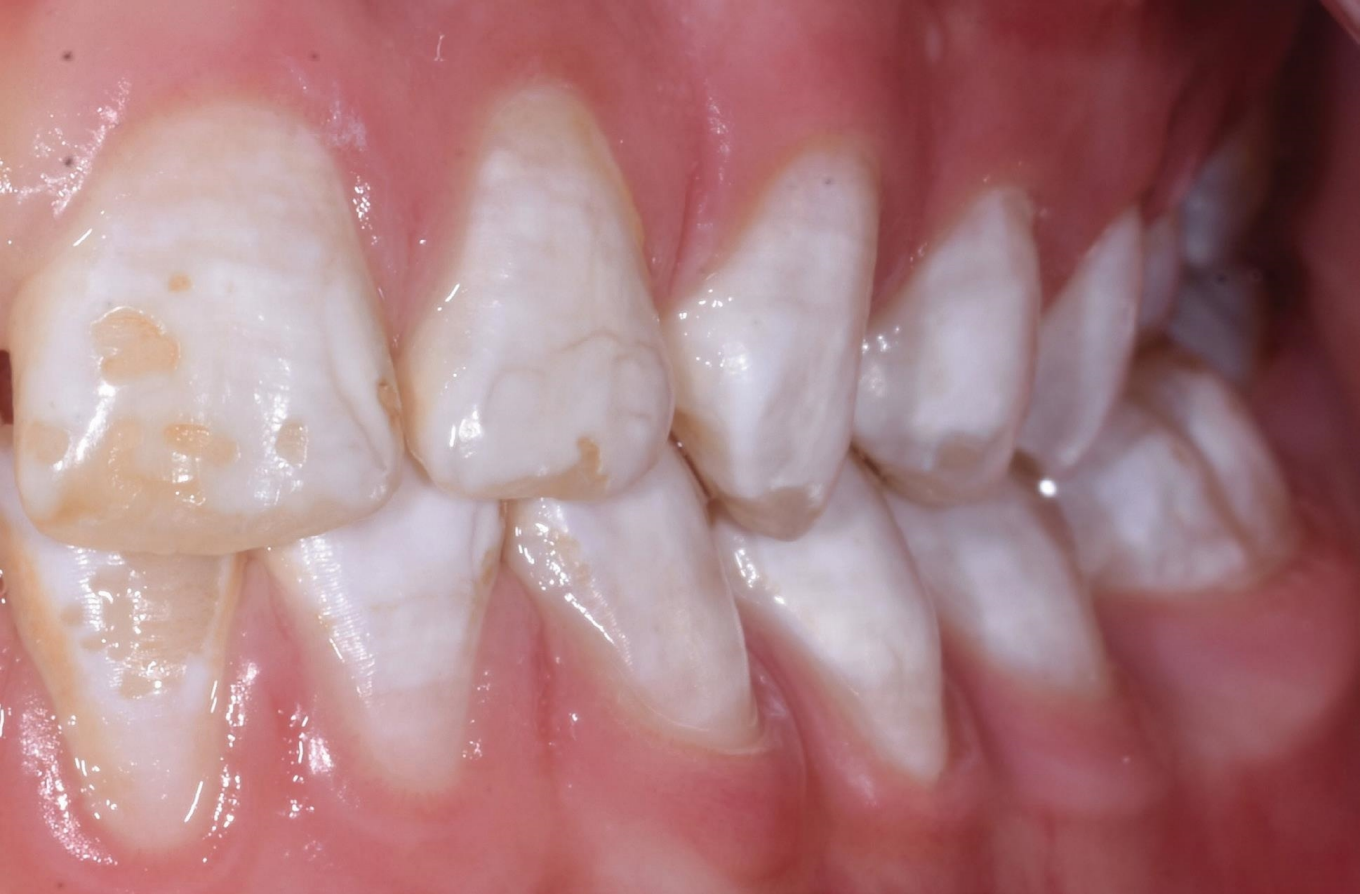
Preoperative intraoral view showing the selection of Tooth 36 for the resin erosion–infiltration protocol.

**Figure 10:**
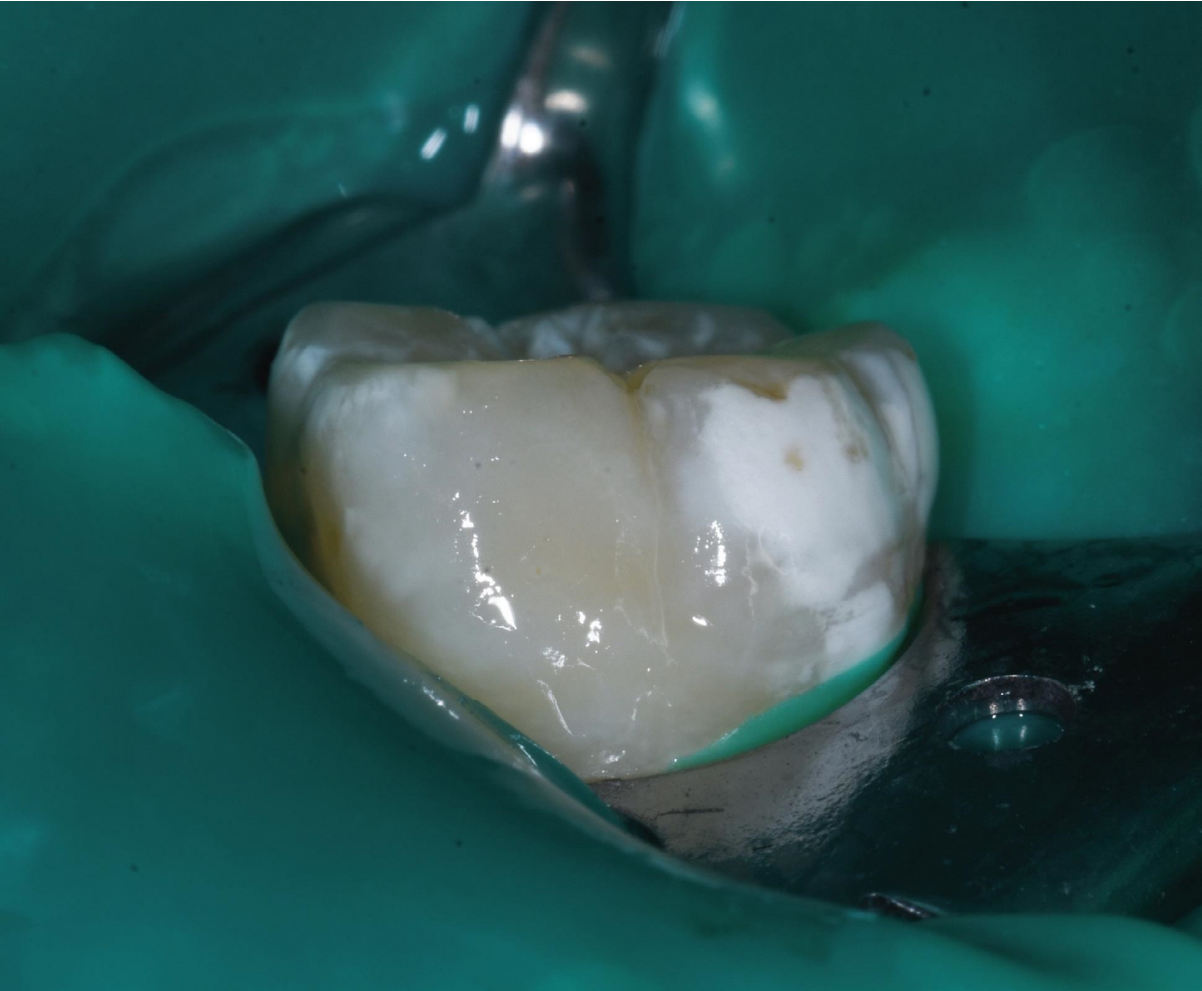
Immediate postoperative view of the mesial half of the buccal surface of Tooth 36 infiltrated.

The erosion‐infiltration technique has proven to be a minimally invasive approach, initially developed for the treatment of early carious lesions and later adapted for anterior hypomineralized stains, such as those seen in molar incisor hypomineralization (MIH) or fluorosis. Since this protocol had not previously been tested for use on hypomature enamel in cases of AI, we first applied it to a less visible tooth (half of the buccal surface of a mandibular first molar) as a preliminary trial (Figure 9). The patient was informed that the treated teeth would appear less white, as the very bright white defects caused by AI contrast with the more translucent appearance of unaffected teeth. Detailed information regarding the episode of care and the clinical procedures performed is provided in Tables S1 and S2, which present, respectively, a chronological timeline of historical and current information and a summary of the treated teeth and associated clinical procedures. As the patient was a minor, her parents were required to sign an informed consent form before treatment began. Two cycles of acid etching (Icon‐Etch, DMG) were performed before proceeding with ethanol (Icon dry, DMG) and resin infiltration (Icon‐Infiltrant, DMG). The result was satisfactory (Figure 10), which led to proceeding with the mandibular anterior teeth.

**Figure 11:**
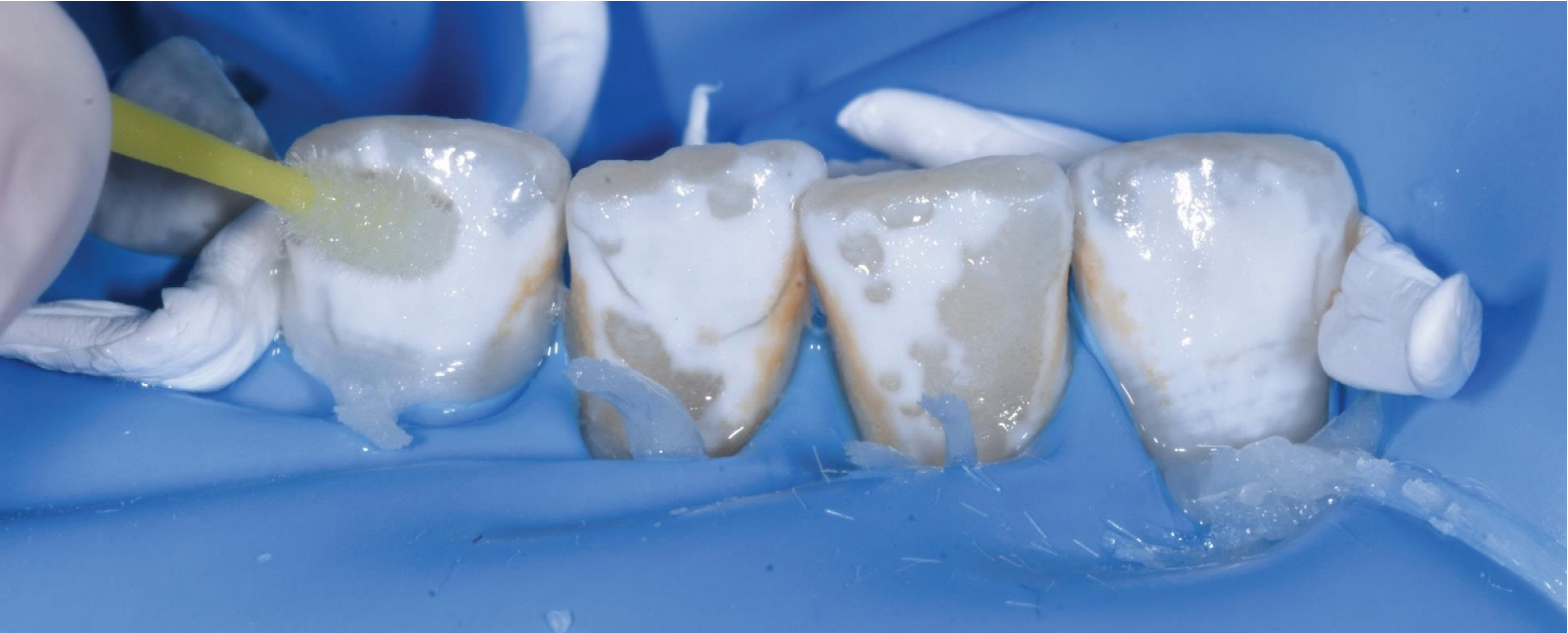
Intraoperative view of deproteinization with sodium hypochlorite for 60 s of mandibular anterior teeth.

The mandibular arch teeth were cleaned, and the operative field was set up. In this case, no local anesthesia was administered, as she did not report any sensitivity related to her hypomature AI, nor during the placement of the clamp for field isolation. Ligatures were placed to enhance isolation and provide better access to the cervical areas of the teeth. Before starting the erosion‐infiltration protocol, the dental surfaces to be treated with resin (Icon, DMG) were deproteinized by rubbing with 5% sodium hypochlorite for 1 min using a microbrush (Figure 11). The teeth were then thoroughly rinsed and completely dried prior to beginning the erosion‐infiltration protocol, which is carried out in three successive steps.

**Figure 12:**
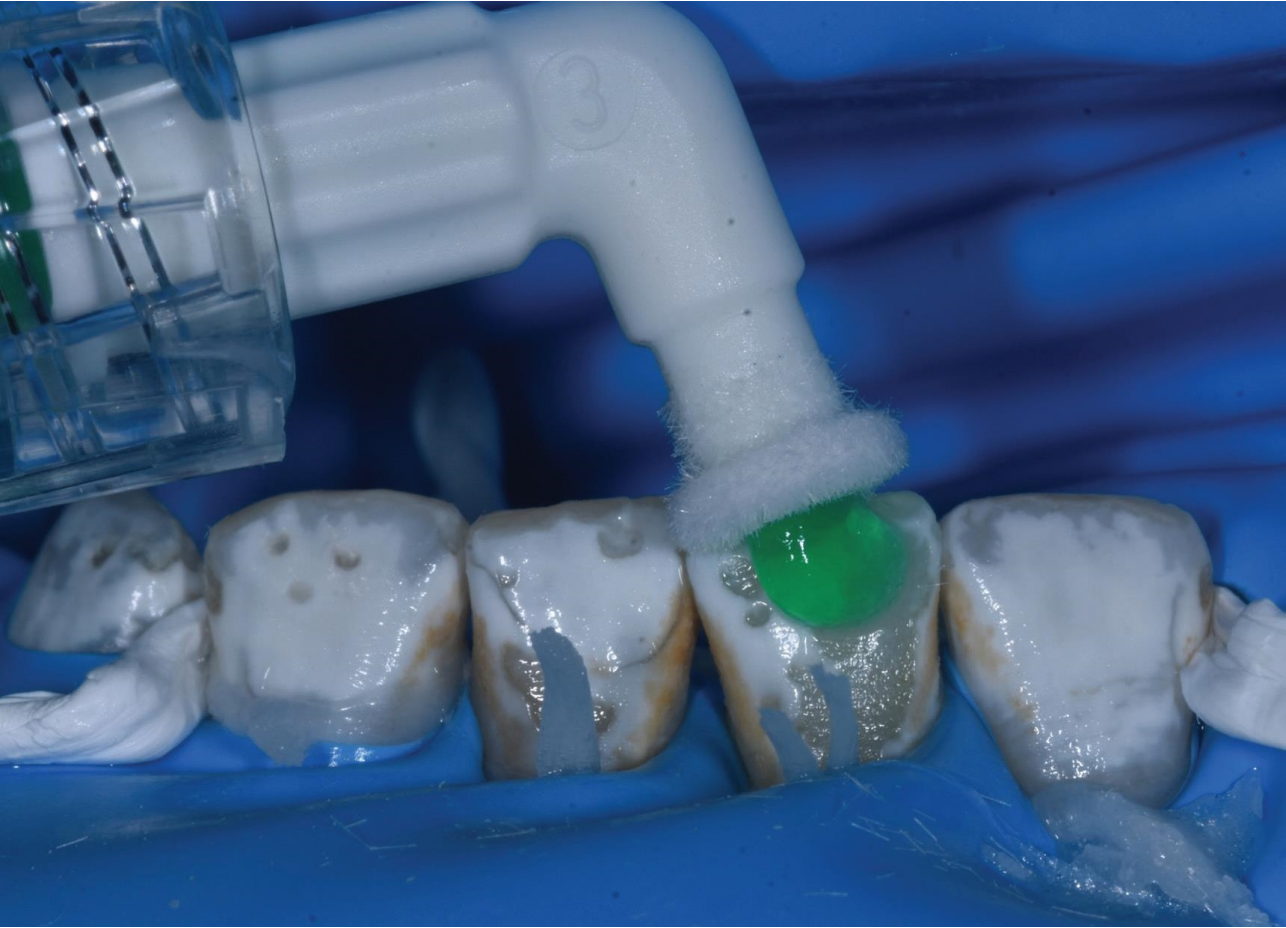
Intraoperative view of application of Icon‐Etch (Icon, DMG) for 2 min on mandibular anterior teeth.

**Figure 13:**
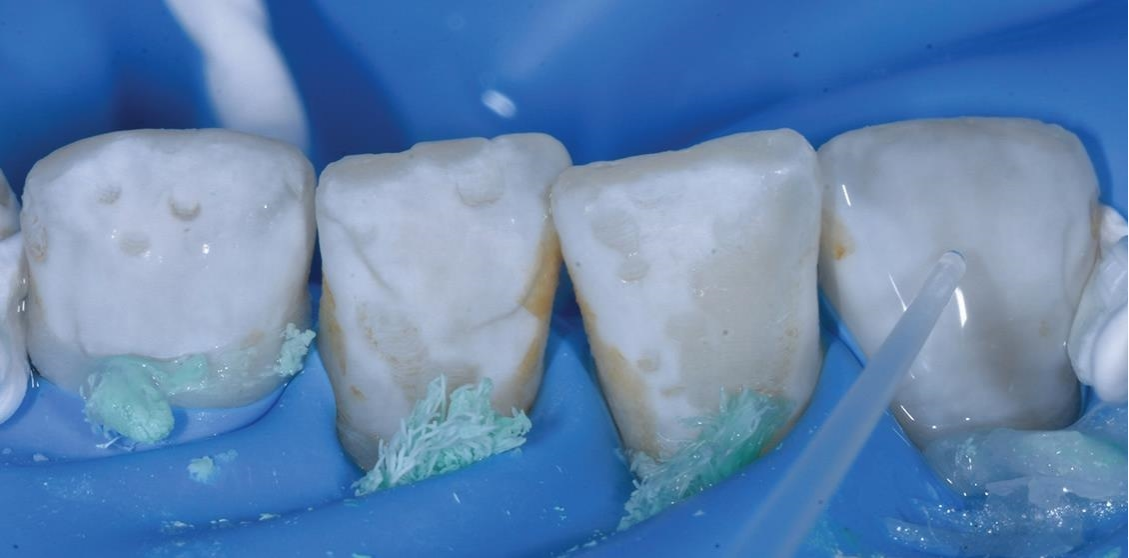
Intraoperative view of application of ethanol for 60 s on mandibular anterior teeth.

**Figure 14:**
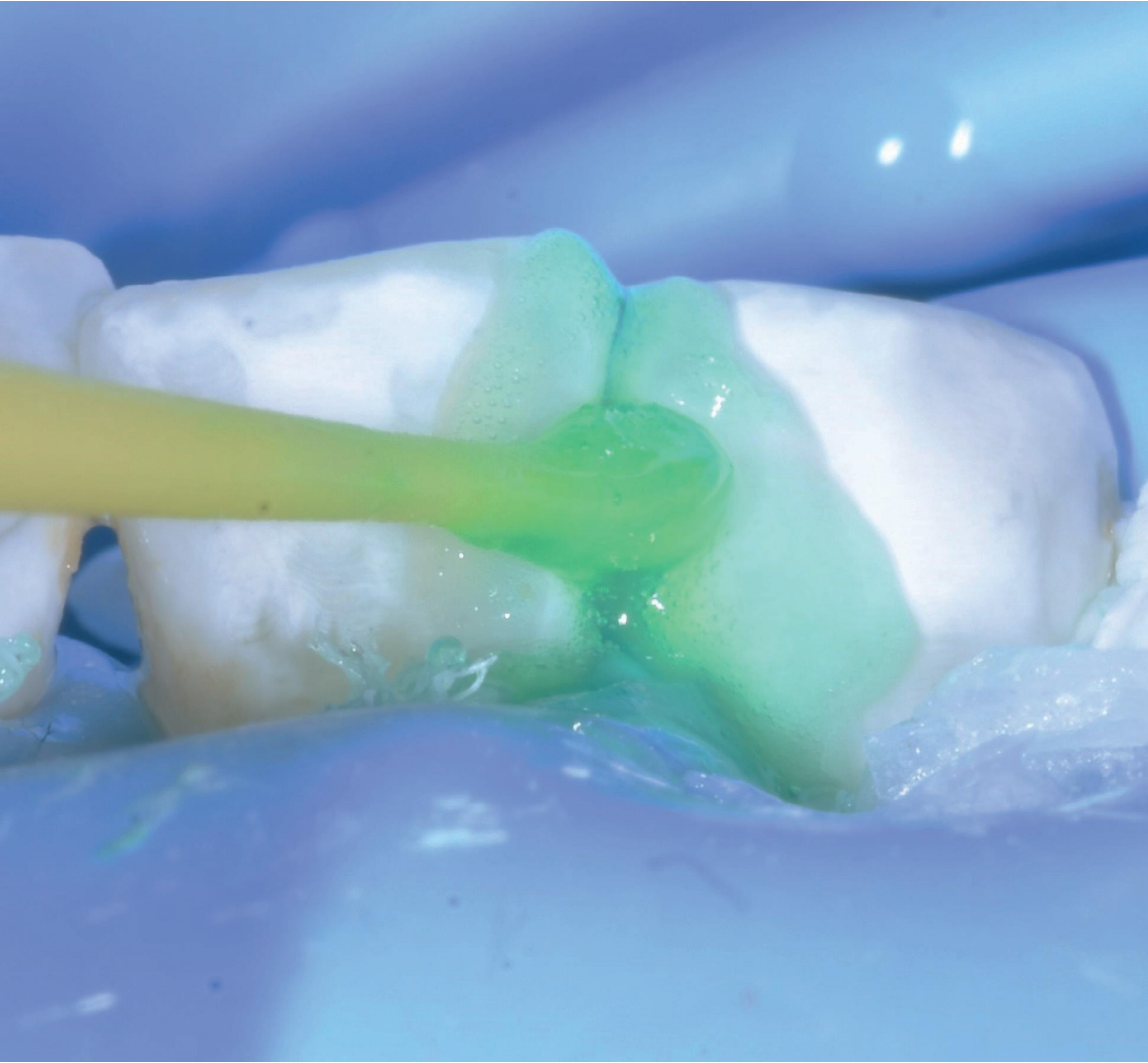
Intraoperative view of second cycle with a new application of hydrochloric acid on residual areas.

Etching was performed by applying Icon‐Etch (Icon, DMG), which contains 15% hydrochloric acid, using the specialized applicator tip provided in the kit. The surface of each tooth to be treated was gently rubbed for 2 min (Figure 12). The treated teeth were then thoroughly rinsed and dried. The drying step was carried out by applying Icon‐Dry (Icon, DMG), which contains 99% ethanol, for 30 s (Figure 13). Ethanol allows a preview of the potential result that may be achieved through resin infiltration, as its optical effect closely resembles that of the resin. Additionally, the alcohol serves to eliminate residual water trapped within the porosities of the lesions. If the result is satisfactory, the next step, resin infiltration with the low‐viscosity resin can be performed. If the defects are not fully masked, a second etching cycle may be carried out. Up to three etching cycles can be performed in total. In this case, the initial etching did not produce a satisfactory result. It was therefore decided to perform a second cycle, limited to the areas that had not responded to the ethanol application (Figure 14). Following the second etching cycle, the hypomature AI defects were adequately masked, allowing the resin infiltration step to proceed.

**Figure 15:**
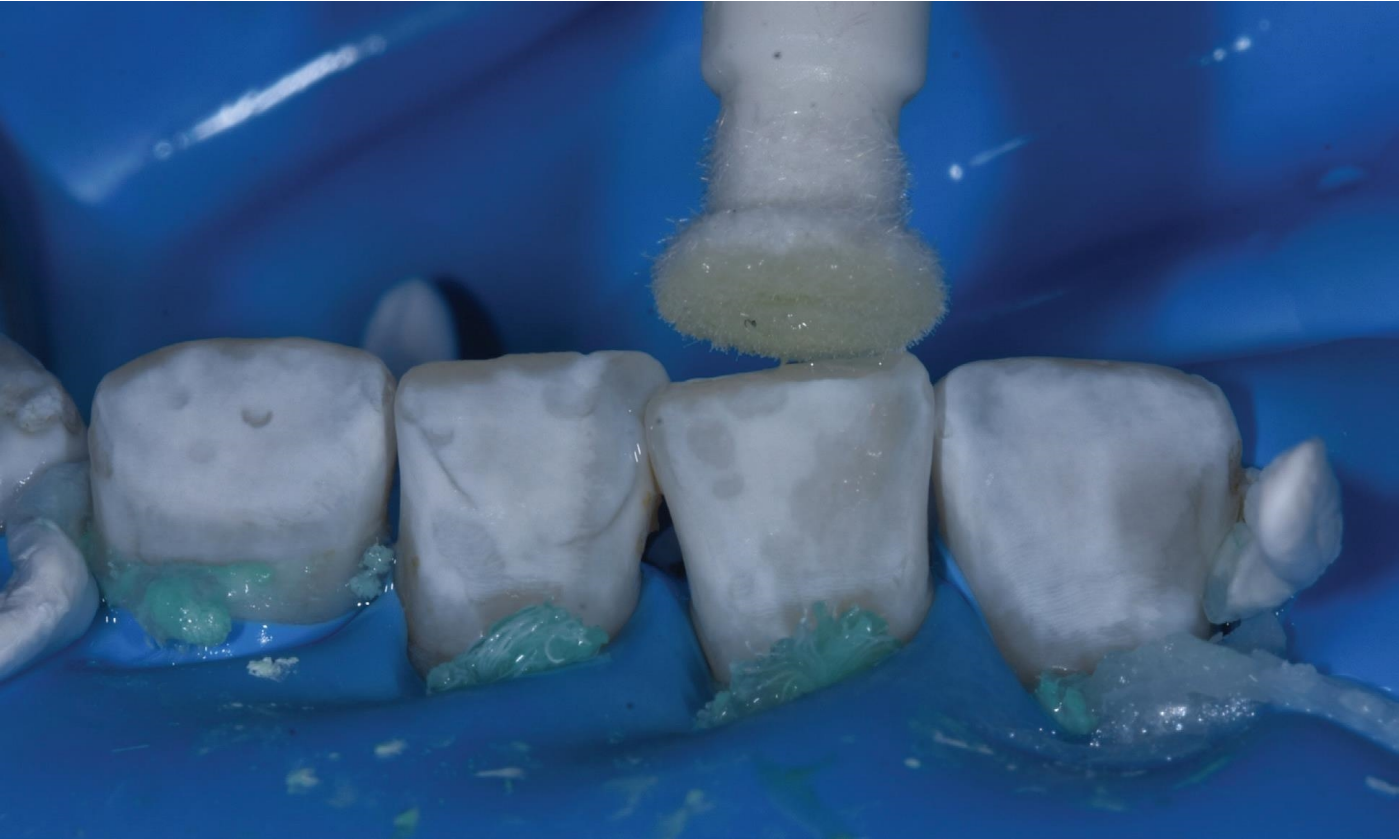
Intraoperative view of infiltration by applying Icon‐Infiltrant (Icon, DMG) for 3 min and initial photopolymerization for 40 s.

**Figure 16:**
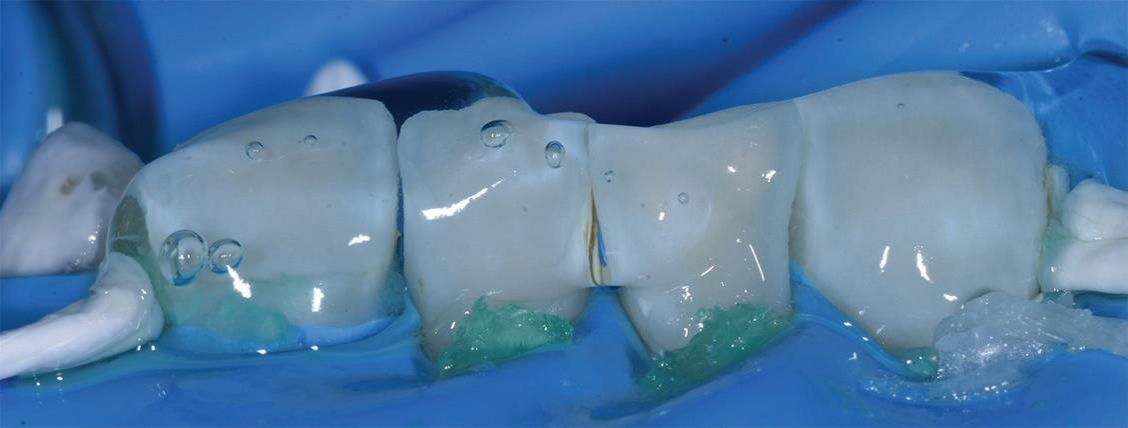
Intraoperative view of final photopolymerization for 40 s under glycerin gel for complete resin setting.

Resin (Icon, DMG) is a low‐viscosity, ultraflowable resin with a refractive index of 1.51. To prevent premature polymerization, the infiltrating resin must be applied out of direct light for 3 min (Figure 15). The resin was then light‐cured for 40 s using a LED curing lamp. A second application of resin (1 min) and 40‐s light‐curing was performed after applying a layer of insulating gel, such as glycerin, over all teeth treated with the infiltrant (Figure 16). The use of glycerin ensures proper polymerization by preventing the resin′s curing inhibition due to oxygen exposure. No additional polishing or finishing procedures were performed after resin infiltration, and no specific postoperative recommendations beyond routine oral hygiene instructions were given.

**Figure 17:**
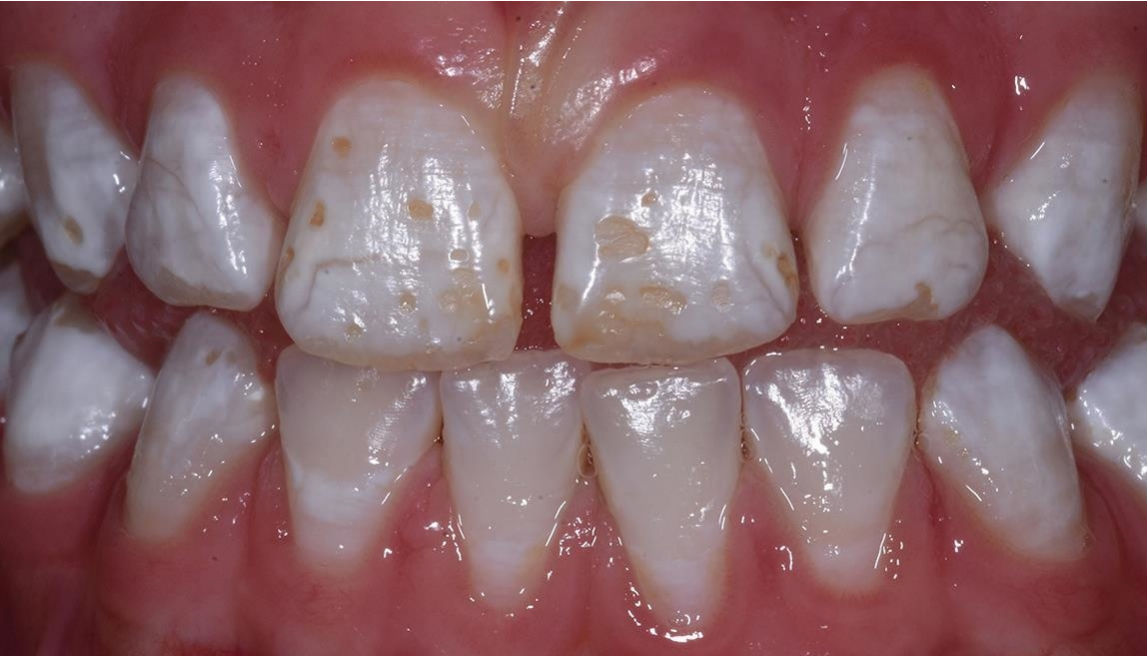
Immediate postoperative view showing the result of infiltration of the mandibular anterior teeth.

Following this first erosion‐infiltration session, the patient was satisfied with the results on her mandibular teeth (Figure 17) and expressed the desire to proceed with treatment on her maxillary teeth during a future session.

**Figure 18:**
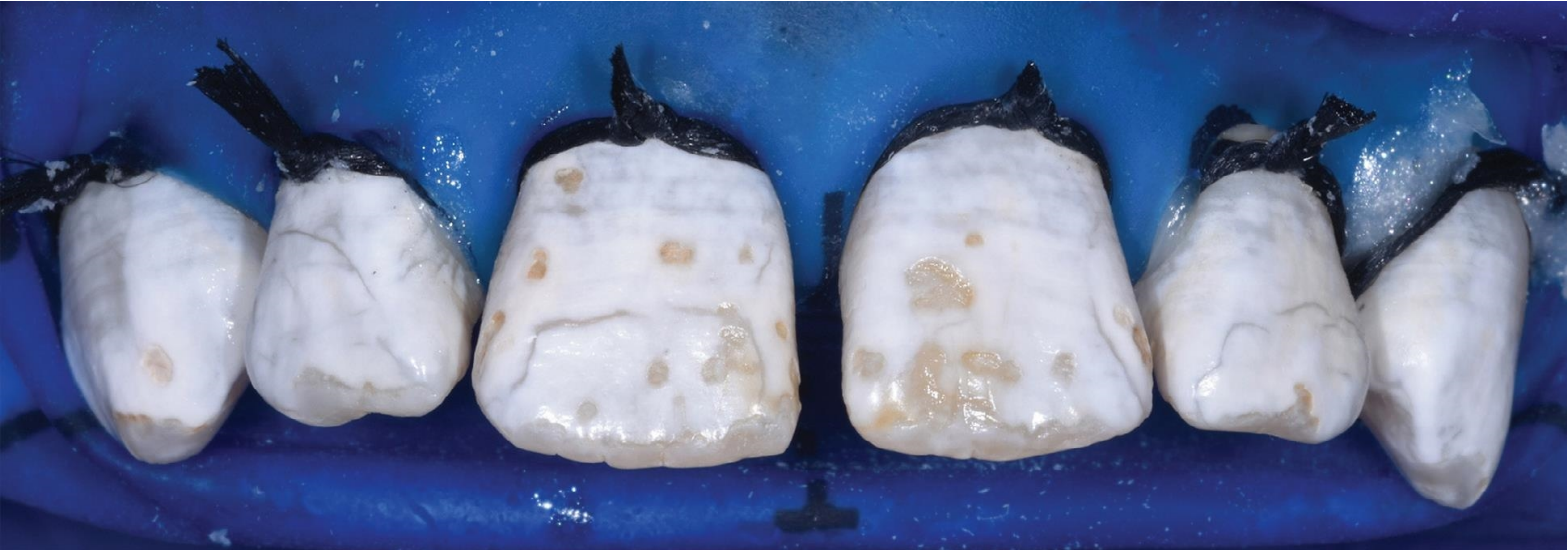
Intraoperative view of isolation of the maxillary anterior teeth using a rubber dam.

**Figure 19:**
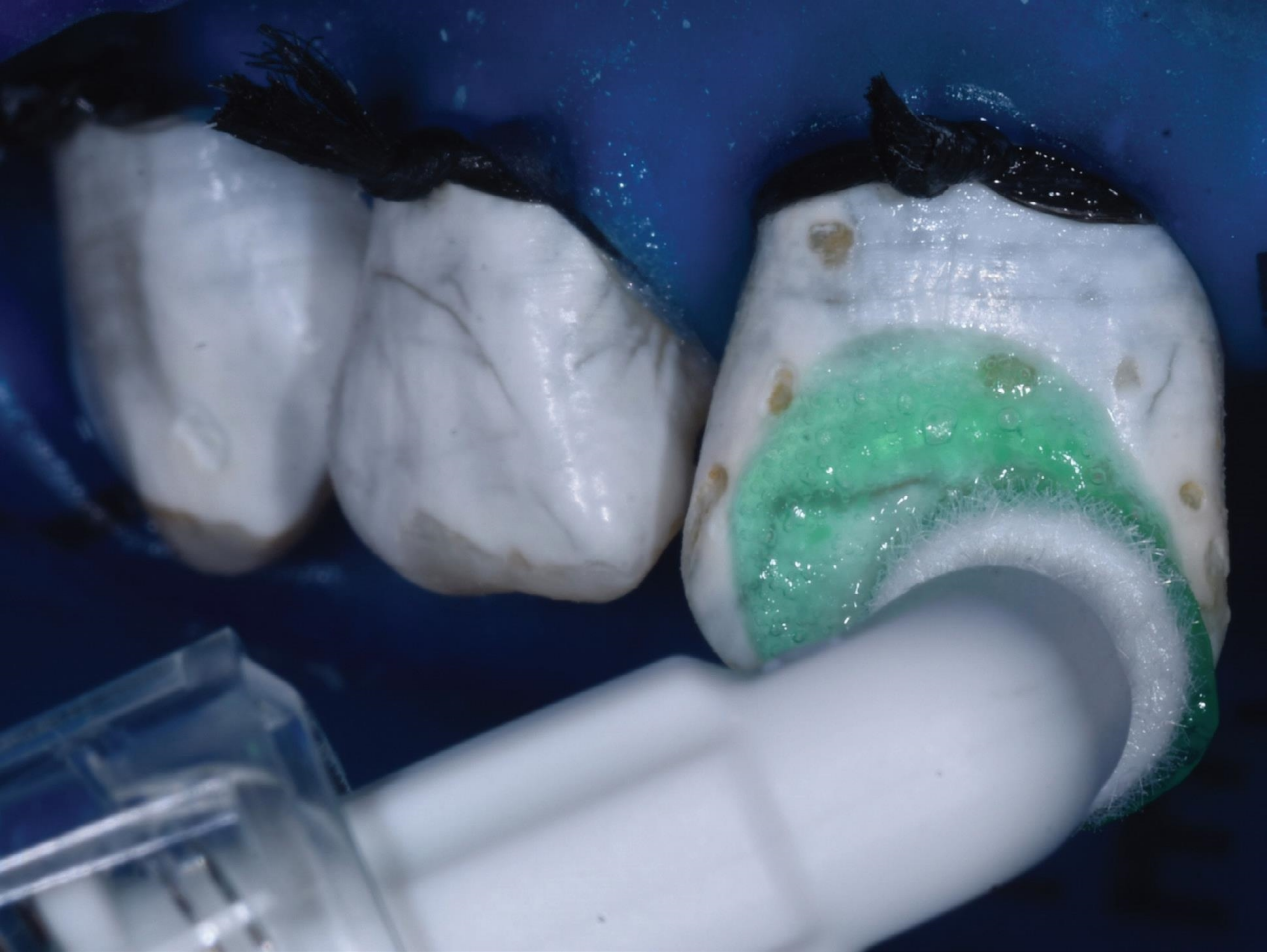
Intraoperative view of application of Icon‐Etch (Icon, DMG) for 2 min on all dental surfaces.

**Figure 20:**
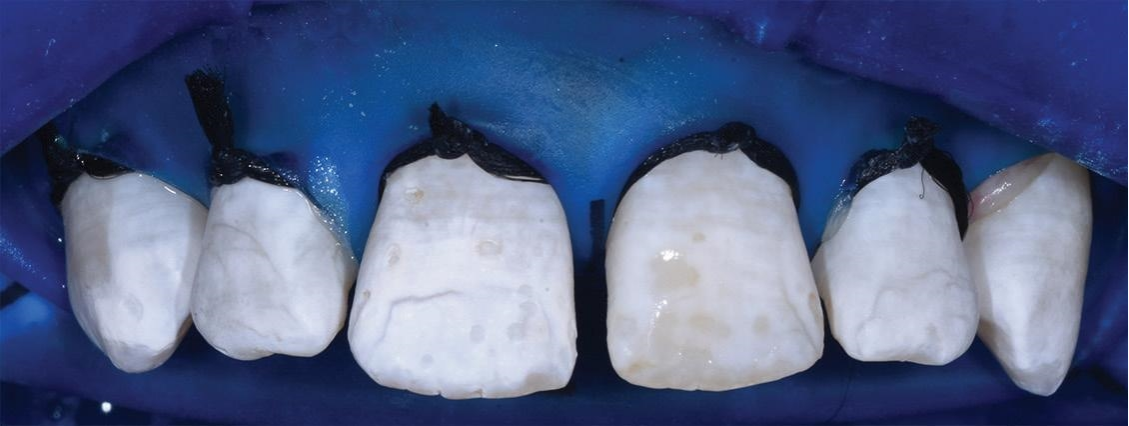
Intraoperative view showing the appearance of dental surfaces before infiltration.

**Figure 21:**
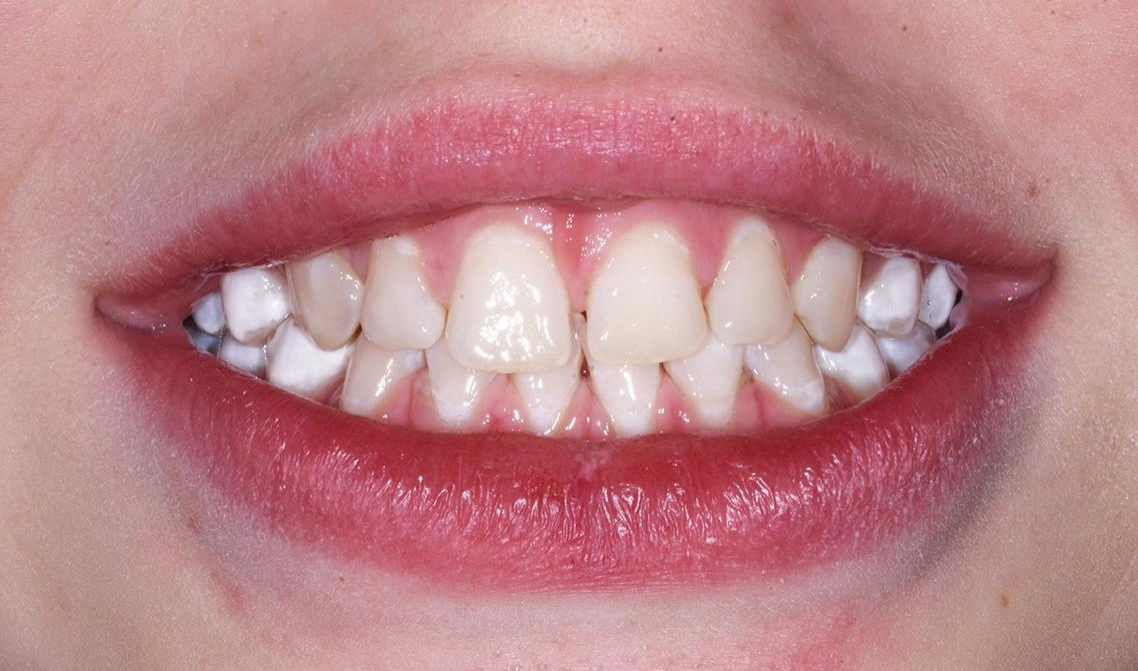
Immediate postoperative view showing the results of infiltration of the maxillary anterior teeth.

The second session was dedicated to the treatment of the anterior teeth in the maxillary arch (Figure 18). The protocol steps were identical, including a maximum of two etching cycles (Figures 19, 20, and 21). Although some areas of teeth required only one etching cycle, others required a second cycle to achieve a satisfactory appearance after ethanol application. In all cases, no more than two etching cycles were performed. The infiltration protocol was otherwise identical for all treated teeth.

**Figure 22:**
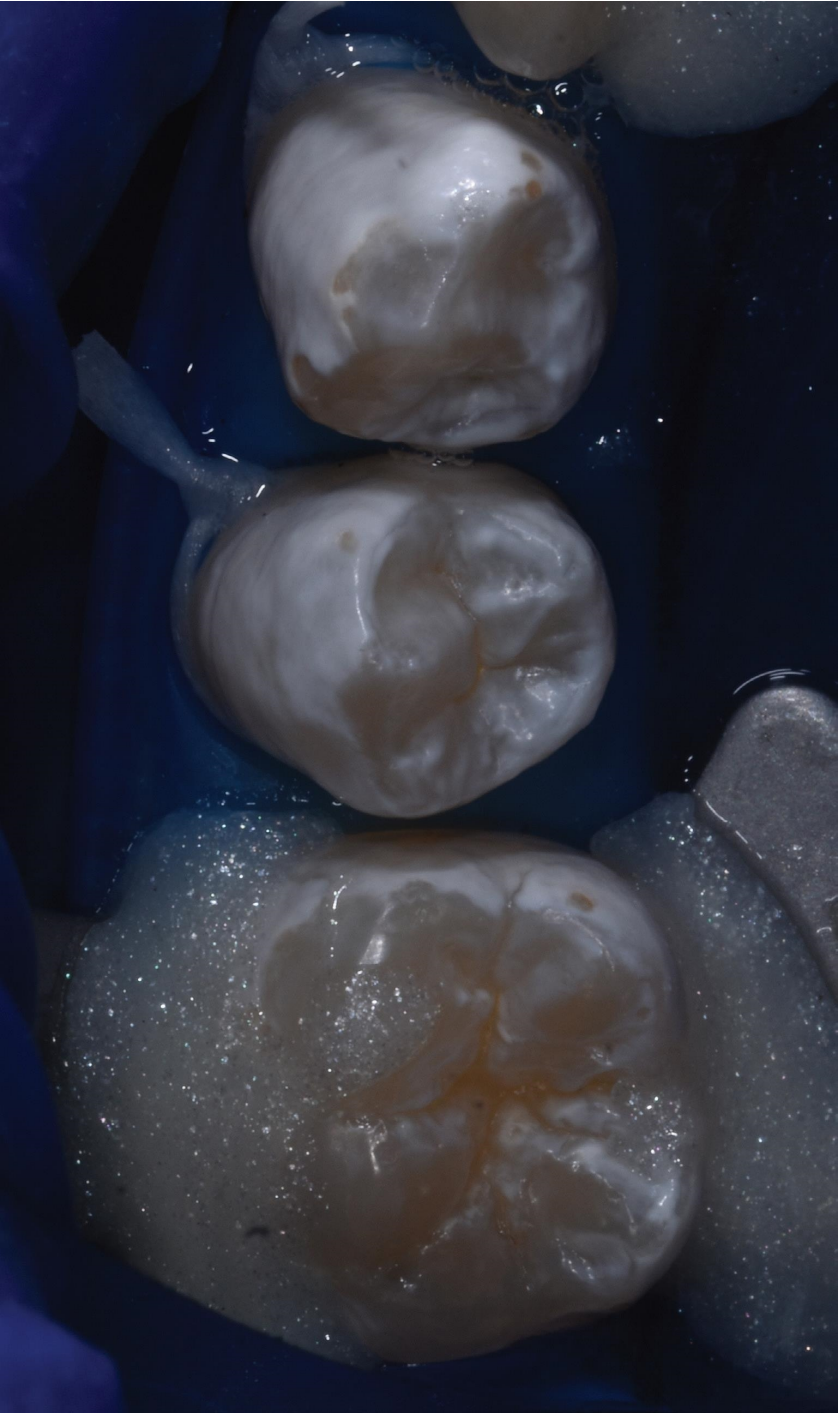
Intraoperative view of isolation of posterior teeth.

**Figure 23:**
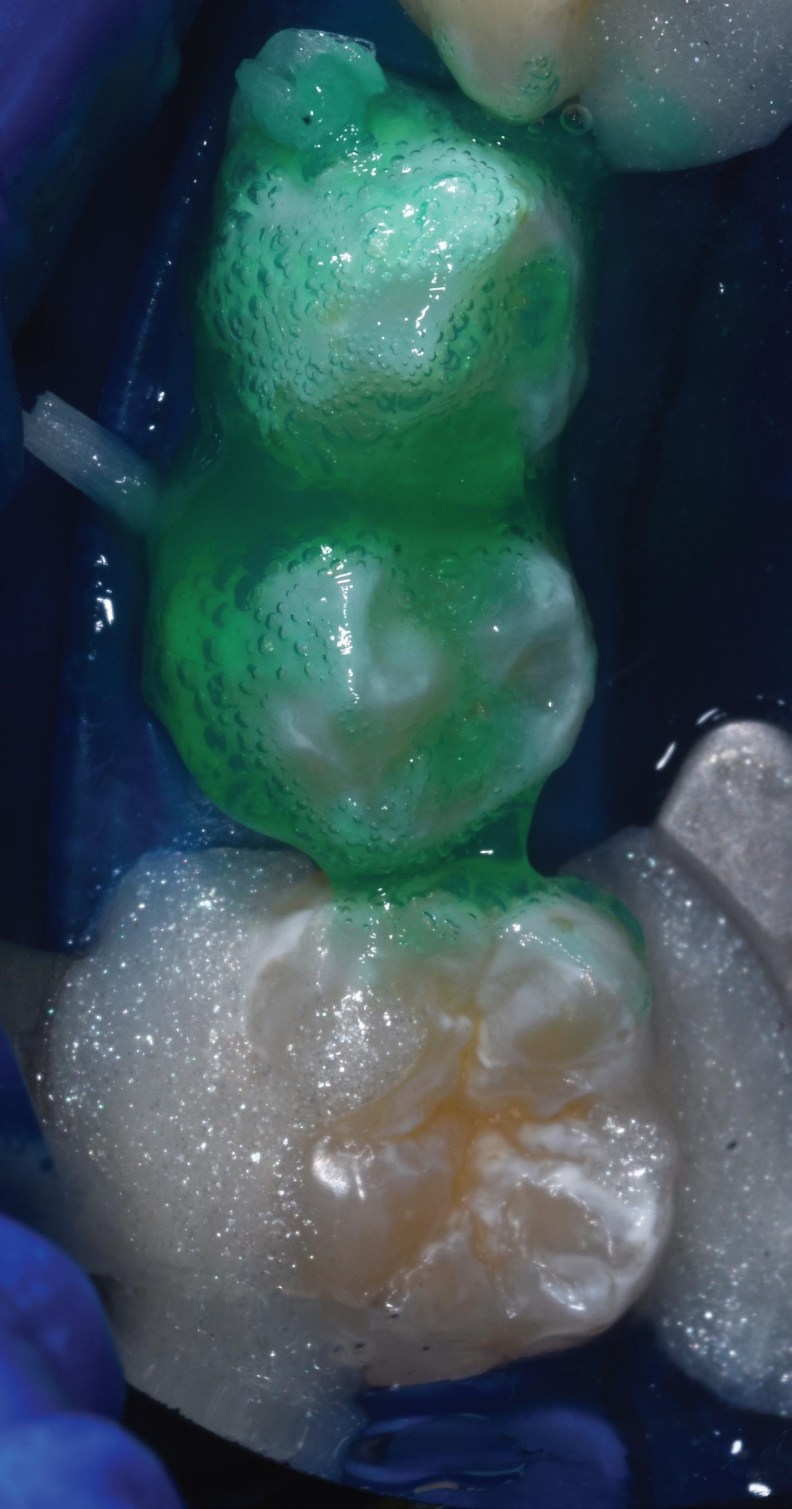
Intraoperative view of application of Icon‐Etch (Icon, DMG) for 2 min on all dental surfaces of the mandibular premolars.

**Figure 24:**
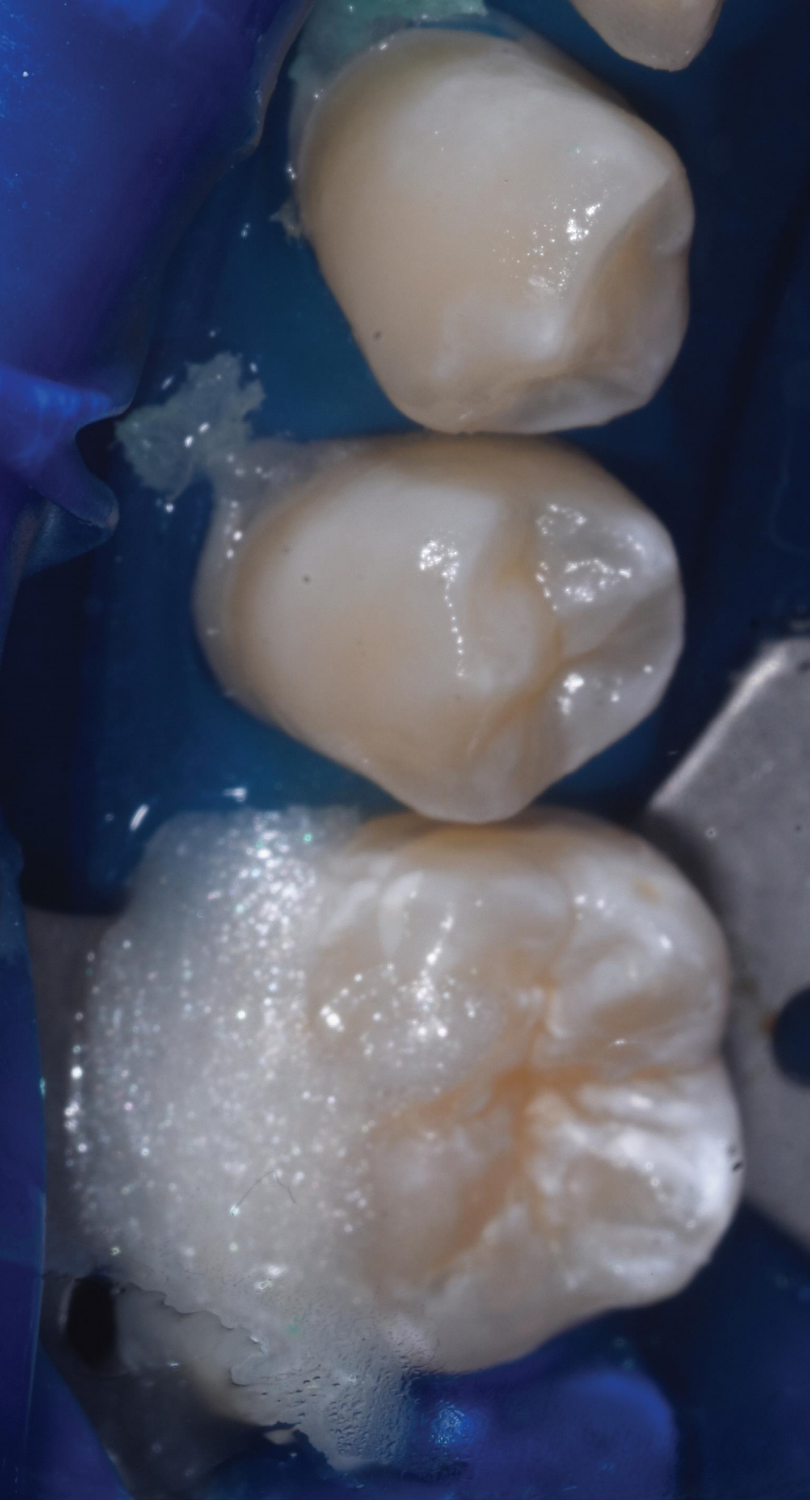
Intraoperative view showing the results of infiltration of Teeth 34 and 35.

**Figure 25:**
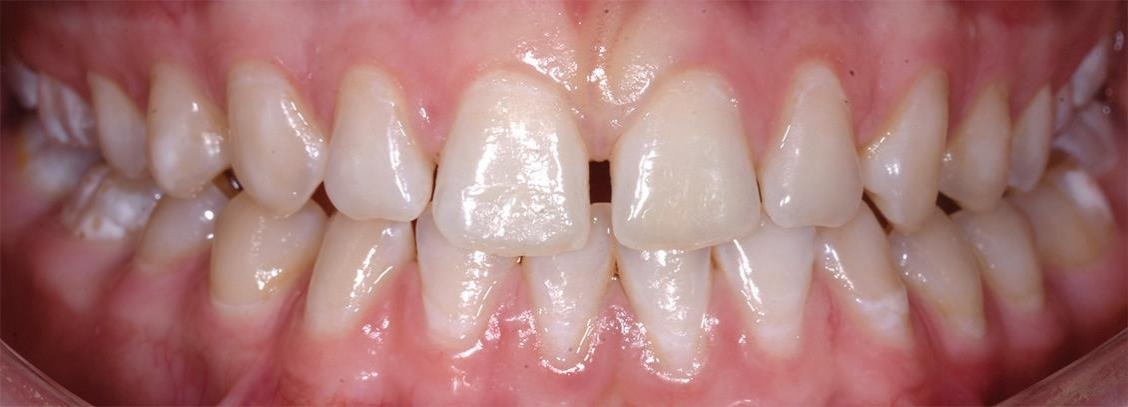
One‐week follow‐up after resin infiltration.

The following two sessions were dedicated to the treatment of the maxillary and mandibular premolars (Figures 22, 23, and 24). The patient was very satisfied with the final result across all treated teeth, which showed complete masking of the hypomature lesions associated with AI (Figure 25). The COHIP‐SF score was 62, indicating good OHRQoL with minimal or no functional, emotional, or social impact.

**Figure 26:**
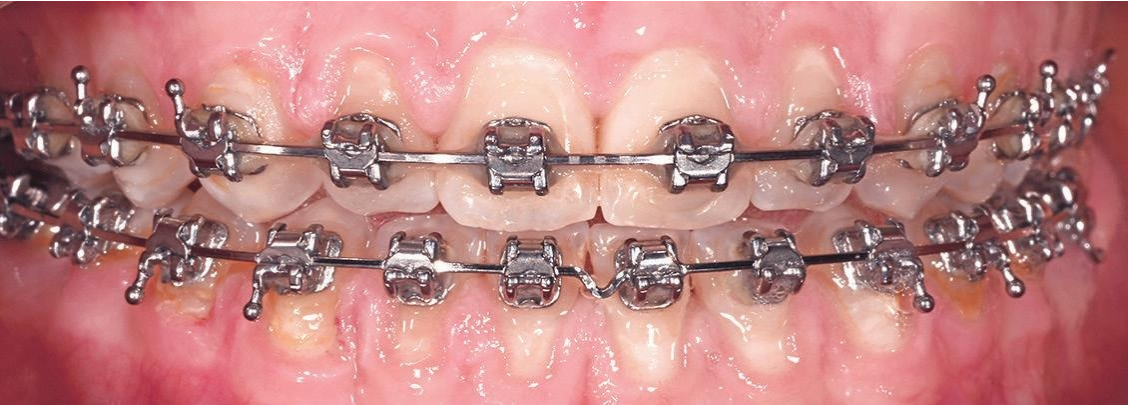
Six‐month follow‐up after resin infiltration. Orthodontic treatment was ongoing.

Six months later, the patient was undergoing orthodontic treatment (Figure 26). No debonding has been reported. Orthodontic bonding is particularly challenging in AI due to reduced bond strength compared with sound enamel [16, 17]. In this case, the creation of a resin‐infiltrated enamel substrate allowed the use of a conventional orthodontic bonding protocol [18], and no bracket debonding was observed.

During the 3‐year follow‐up, no retreatment or additional polishing was required. The infiltrated surfaces remained clinically stable, without marginal deterioration, surface wear, or clinically significant discoloration. During orthodontic treatment, the patient attended regular 6‐month recall visits for professional prophylaxis and topical fluoride varnish application.

**Figure 27:**
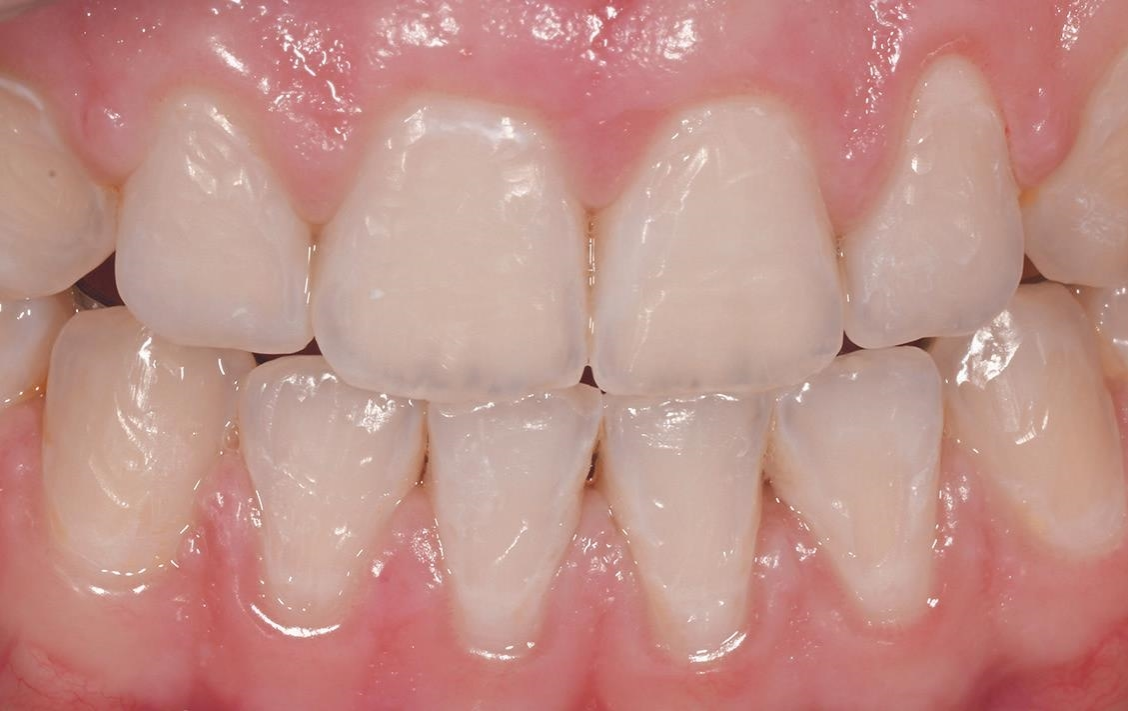
Three‐year follow‐up after resin infiltration.

Three years later, the orthodontic treatment has been completed, and the patient remained very satisfied with the appearance of her teeth (Figure 27).

## 3. Discussion

The enamel anomalies caused by hypomature AI lead to a significant esthetic burden. The objective of this clinical case was to assess the effectiveness of resin infiltration in restoring satisfactory esthetics in a case of hypomature AI. In our case, the patient was highly satisfied with the technique and the final result. It allowed her to smile again after years of trying to hide her teeth due to the AI lesions, which had made her the target of teasing. AI is associated with significant impairment of OHRQoL in children and adolescents. In this case, resin infiltration was associated with a substantial improvement in COHIP‐SF scores, reflecting not only enhanced esthetics but also significant psychosocial benefits. These findings highlight the relevance of conservative esthetic management strategies in addressing both clinical and patient‐reported outcomes. However a limitation of the present case report is the absence of objective esthetic assessment methods, such as spectrophotometric measurements, colorimetric analyses, or standardized esthetic indices. Consequently, the esthetic outcome was primarily evaluated through clinical examination, standardized photographic documentation, patient‐reported satisfaction, and the improvement observed in the COHIP‐SF score. Although these complementary assessments consistently indicated a favorable esthetic and psychosocial outcome, the lack of objective quantitative measurements should be considered when interpreting the results. Future studies including standardized instrumental esthetic evaluations would provide more robust evidence regarding the effectiveness of resin infiltration in patients with AI.

To date, a systematic review on the management of AI in children and adolescents only reports treatment options ranging from composite resin restorations to ceramic crowns. Minimally invasive approaches should be fully recognized as valid therapeutic options [8]. Given the favorable outcomes observed with resin infiltration in AI lesions and its tissue‐preserving nature, resin infiltration (Icon, DMG) may be considered a promising minimally invasive treatment option before more invasive restorative approaches, such as porcelain veneers, in selected cases [19, 20]. However, further prospective clinical studies are needed to confirm its efficacy and long‐term outcomes before it can be recommended as a first‐line treatment. Cagetti et al. [21] are aimed at evaluating the esthetic effectiveness of a noninvasive approach for AI. Two cases of “supposed hypomature AI” were treated with low‐viscosity resin. However, the clinical presentation described in these two cases may also be consistent with dental fluorosis, given the symmetrical distribution of lesions, their confinement to incisal areas, and the absence of involvement of all teeth. Rogers et al. [22] treated a 13‐year‐old girl with hypomature AI sought treatment for tooth discoloration affecting her social interactions; microabrasion using 6.6% hydrochloric acid was performed on Teeth 11, 12, and 13. The following day, the patient′s father reported that her teeth had turned orange, attributed to eating a tomato pizza immediately after treatment. The extrinsic staining was successfully removed using 16% carbamide peroxide in a custom night tray over 2 weeks. Ten cycles of microabrasion were completed per tooth, each cycle lasting 60 s and followed by washing and drying, polishing with a fine polishing disc and application of sodium fluoride. However, minimal esthetic improvement was noted, with only a slight reduction in the visibility of the opacities. Subsequently, direct composite resin veneers were placed on the maxillary anterior teeth, resulting in an optimal esthetic outcome, and the patient remained satisfied with her dental appearance. This technique proves to be more invasive, and treatment with Icon could have been considered. As this treatment removes only a minimal amount of enamel, no veneers are subsequently required, thereby avoiding the maintenance associated with these temporary restorations.

Erosion‐infiltration technique may also potentially help patients with AI by reducing dental hypersensitivity. Although no clinical studies have specifically evaluated resin infiltration for hypersensitivity management in AI, evidence from MIH is encouraging. Parajón‐Oliveros et al. [23] reported a significant reduction in hypersensitivity (VAS score from 8 to 0 over a 24‐month follow‐up), together with excellent esthetic and clinical outcomes after resin infiltration of anterior teeth affected by MIH. Given the increased enamel porosity and permeability shared by both MIH and AI, resin infiltration may reduce enamel permeability and limit stimulus transmission, making it a promising minimally invasive approach for managing sensitivity in AI. However, clinical studies are needed to confirm its effectiveness in this population.

A systematic review demonstrates that infiltrative resins effectively change the properties of demineralized enamel [24]. Regarding white spot lesions, infiltrative resins reduced enamel surface roughness but increased its microhardness and shear bond strength. Furthermore, estimates point to an average penetration depth capacity of 65% by this type of resin [24]. In hypomineralized enamel due to MIH, microhardness increased in areas of resin penetration by 1.0 ± 0.7 GPa representing a proportional increase of 2.2 ± 2.5 times [25]. However, it should be noted that the depth of resin infiltration in hypomineralized enamel can be heterogeneous, presenting challenges in achieving consistent and complete infiltration throughout the lesion [25]. Although erosion‐infiltration has been used for several years to treat white enamel lesions such as MIH and fluorosis, it is not considered a standard treatment option for AI. The enamel of hypomature AI shows numerous cracks, lamellae, and an increased number of tufts [26]. This hypomature enamel also showed increased organic matrix retention, higher carbonate content, reduced crystallinity, and smaller hydroxyapatite crystal size [27]. The findings highlight the critical role of organic matrix removal during enamel maturation in achieving proper mineralization. As MIH, one of the main challenges in AI treatment is achieving strong adhesion to enamel, which is compromised by the higher protein content. Infiltration by low‐viscosity resin (Icon, DMG), effective in the management of MIH‐affected enamel, can legitimately be applied to AI. This hypomineralized enamel due to MIH makes it less favorable for bonding due to the protein level in MIH enamel that is 8–21 times higher than in sound enamel [28]. Clinical investigations have explored pretreatment with 5% sodium hypochlorite and the use of self‐etch or total‐etch techniques [29]. Sodium hypochlorite is an oxidizing agent and degrades protein. For developmental enamel defects, a systematic review recommended performing bonding on enamel using an etch‐and‐rinse technique in cases of fluorosis and MIH [30]. Alternative strategies, although supported by weaker evidence, involve enamel deproteinization in cases of fluorosis, MIH, and AI, along with modifications of etching parameters for fluorosis and AI [30]. The optimal sequence of deproteinization and acid etching in teeth affected by AI remains controversial. A recent systematic review concluded that there is only a very low level of evidence supporting the use of a deproteinization step with 5% sodium hypochlorite to improve adhesive performance in AI‐affected enamel [30]. Conversely, pre‐etching application of sodium hypochlorite has been shown to increase the depth of acid penetration by facilitating the removal of organic components from the enamel surface. In the present case, the protocol described by Amend et al. [31], involving sodium hypochlorite application before acid etching, was followed. Given the limited and conflicting evidence currently available, further clinical studies are needed to establish the optimal sequence of deproteinization and acid etching in patients with AI.

In the conventional resin infiltration protocol (Icon, DMG), ethanol is primarily used as a drying agent to remove residual moisture from the porous enamel before resin application. Although the temporary masking effect produced by ethanol may provide an indication of the expected esthetic result, this is not its primary purpose. As highlighted by Marouane and Chtioui [32], transillumination represents an alternative diagnostic approach that allows assessment of lesion depth, monitoring of enamel layer removal, and evaluation of resin infiltration progression, thereby improving the predictability of the procedure without relying on ethanol as an esthetic preview. This treatment protocol could also have been considered.

The erosion‐infiltration treatment offers many advantages, but also some limitations. It is quick and simple, and often can be completed in a single session if few teeth or a single arch are treated. Its painlessness makes it ideal for anxious patients. Although anesthesia is recommended for placing the rubber dam, it is not essential, especially if no microabrasion or drilling is needed. Erosion‐infiltration also respects the therapeutic gradient, being minimally invasive and preserving healthy tissue. For superficial lesions, microabrasion is not necessary. However, the procedure is expensive. Thus, a cost estimate and informed consent must be provided before treatment. For deeper lesions, partial enamel removal by sandblasting or drilling may be required to reach the lesion′s core and improve infiltration, followed by direct restoration such as composite layering, thus increasing treatment time.

Treated teeth must be isolated with a rubber dam throughout the procedure to protect soft tissues from Icon‐Etch, which can cause burns, and to ensure proper resin polymerization, which is hydrophobic. If the dam alone does not provide full isolation, liquid dam should be added [33].

An in vitro study evaluated the biocompatibility of TEGDMA resin (Icon, DMG), specifically its effects on dental pulp stem cells. Cells exposed to polymerized resin showed low cytotoxicity (~10%), whereas those in contact with nonpolymerized Icon (DMG) had high cytotoxicity (~98.9%). Various TEGDMA concentrations (the main component of Icon, DMG) were tested, revealing a dose‐dependent increase in cytotoxicity [34]. Another study indicated that the most harmful effects were likely due to hydrochloric acid in Icon‐Etch (Icon, DMG) [35].

Due to the recent introduction of this procedure, long‐term clinical data are lacking, making it difficult to predict the material′s aging. However, a systematic review evaluated the postoperative stability of esthetic results after resin infiltration in hypomineralized and demineralized permanent teeth. Follow‐up ranged from 6 months to 6 years, and results, measured by resin colorimetry, were consistently stable in the short, medium, and long term [36].

Long‐term follow‐up is needed to determine whether this approach is a reliable and effective treatment for enamel discoloration caused by AI. Strict adherence to the manufacturer′s protocol is also essential to avoid potential adverse effects. As this was the first application of resin infiltration for the management of AI in this young patient, we elected to first evaluate the immediate clinical and esthetic outcome on a posterior tooth before extending the procedure to the remaining dentition, particularly the anterior teeth. For each new patient, we repeat this preliminary assessment on a posterior tooth before proceeding with treatment of the remaining dentition.

## 4. Conclusion

The esthetic management of hypomature AI should follow a minimally invasive therapeutic gradient and adapt to evolving clinical techniques. Currently, there is no standardized treatment protocol for AI, and the therapeutic approach is determined on a case‐by‐case basis, depending on the patient′s needs and preferences. The erosion‐infiltration technique using Icon may represent a relevant conservative option that can be implemented at an early stage, prior to resorting to irreversible restorative procedures.

In the present case, resin infiltration has proven to be a minimally invasive and highly satisfactory esthetic treatment for white enamel opacities related to AI, offering a gentler alternative to conventional methods. This approach may be recommended for achieving long‐lasting esthetic improvement, particularly in children and adolescents. This treatment also has the advantage of enhancing bracket adhesion to enamel during orthodontic treatment and reducing the risk of white spot lesions.

However, the long‐term durability of resin infiltration in AI remains insufficiently documented. Although esthetic results appeared stable in this case, longer follow‐up periods and larger clinical series are required to provide stronger evidence of the treatment′s durability. Furthermore, since this represents one of the first uses of low‐viscosity resin infiltrant for treating enamel defects caused by AI, it cannot yet be considered a reference treatment for this condition. Protocol adjustments may also be necessary in the future. The encouraging results observed in this case should be considered preliminary. Larger case series with long‐term follow‐up, and ultimately prospective controlled clinical studies, are needed to better establish the effectiveness, durability, and indications of resin infiltration in patients with hypomature AI.

## Author Contributions

E.G.: conceptualization, investigation, methodology, data curation, visualization, writing—original draft, and writing—review and editing. J.E., A.C., A.R., O.K., and J.V.G.: writing—review and editing.

## Funding

No funding was received for this manuscript.

## Disclosure

All authors have read and approved the final version of the manuscript. The corresponding author had full access to all of the data in this study and takes complete responsibility for the integrity of the data and the accuracy of the data analysis.

## Ethics Statement

The ethical approval was not required due to single anonymized case report with informed consent.

## Conflicts of Interest

The authors declare no conflicts of interest.

## Supporting Information

Additional supporting information can be found online in the Supporting Information section.

## Supporting information


**Supporting Information 1** Table S1: Historical and current information from this episode of care organized as a timeline. Table S2: Summary of treated teeth and clinical procedures.

**Supporting Information 2**  

## Data Availability

The data that support the findings of this study are available from the corresponding author upon reasonable request.
