# Supplementary material for: Resin Infiltration for the Management of Hypomature Amelogenesis Imperfecta: A Case Report
Source: Case Rep Dent. 2026 Sep 2;2026:7795936. doi: 10.1155/crid/7795936 (PMC13539065; doi:10.1155/crid/7795936)
Supplement: Supplementary file 1 [file CRID-2026-7795936-s002.pdf]

CARE case report guidelines

CARE Checklist of information to include when writing a case report

(cc) BY-NC-ND

Topic	Item	Checklist item description	Reported on Line
Title	1	The diagnosis or intervention of primary focus followed by the words "case report"	1
Key Words	2	2 to 5 key words that identify diagnoses or interventions in this case report, including "case report"	48-49
Abstract (no references)	3a	Introduction: What is unique about this case and what does it add to the scientific literature?	34-38
	3b	Main symptoms and/or important clinical findings	26
	3c	The main diagnoses, therapeutic interventions, and outcomes	27-28
	3d	Conclusion—What is the main "take-away" lesson(s) from this case?	40-46
Introduction	4	One or two paragraphs summarizing why this case is unique (**may include references**)	122-130
Patient Information	5a	De-identified patient specific information.	141
	5b	Primary concerns and symptoms of the patient.	141-143
	5c	Medical, family, and psycho-social history including relevant genetic information	173-179
	5d	Relevant past interventions with outcomes	NONE relevant
Clinical Findings	6	Describe significant physical examination (PE) and important clinical findings.	155-165
Timeline	7	Historical and current information from this episode of care organized as a timeline	supplementary information
Diagnostic Assessment	8a	Diagnostic testing (such as PE, laboratory testing, imaging, surveys).	164-165
	8b	Diagnostic challenges (such as access to testing, financial, or cultural)	87-89
	8c	Diagnosis (including other diagnoses considered)	166-179
	8d	Prognosis (such as staging in oncology) where applicable	Not applicable
Therapeutic Intervention	9a	Types of therapeutic intervention (such as pharmacologic, surgical, preventive, self-care)	180-181
	9b	Administration of therapeutic intervention (such as dosage, strength, duration)	188-241
	9c	Changes in therapeutic intervention (with rationale)	Not applicable
Follow-up and Outcomes	10a	Clinician and patient-assessed outcomes (if available)	242-245
	10b	Important follow-up diagnostic and other test results	246-247
	10c	Intervention adherence and tolerability (How was this assessed?)	235-236
	10d	Adverse and unanticipated events	Not applicable
Discussion	11a	A scientific discussion of the strengths AND limitations associated with this case report	249-378
	11b	Discussion of the relevant medical literature **with references**.	249-378
	11c	The scientific rationale for any conclusions (including assessment of possible causes)	381-402
	11d	The primary "take-away" lessons of this case report (without references) in a one paragraph conclusion	387-392
Patient Perspective	12	The patient should share their perspective in one to two paragraphs on the treatment(s) they received	246-247
Informed Consent	13	Did the patient give informed consent? Please provide if requested	Yes ☒ No ☐
