# Supplementary material for: Resin Infiltration for the Management of Hypomature Amelogenesis Imperfecta: A Case Report
Source: Case Rep Dent. 2026 Sep 2;2026:7795936. doi: 10.1155/crid/7795936 (PMC13539065; doi:10.1155/crid/7795936)
Supplement: Supplementary file 2 [file CRID-2026-7795936-s001.docx]

| **Time** | **Clinical history / Assessment** | **Intervention** | **Outcome** |
| --- | --- | --- | --- |
| **Initial consultation (Age 11 years)** | White, generalized enamel opacities affecting both primary and permanent dentitions. Esthetic concerns, teasing at school, reduced self-esteem. Similar phenotype observed in the younger sister, suggesting hereditary AI.  Clinical examination, panoramic radiograph, differential diagnosis (hypomature AI vs fluorosis), COHIP-SF score = **28**, indicating severe impairment of oral health-related quality of life. | Diagnosis of generalized hypomature amelogenesis imperfecta. Treatment options discussed with the patient and parents. Written informed consent obtained. | Progressive psychosocial impact.  Minimally invasive resin infiltration selected. |
| **Treatment session 1** | Preliminary evaluation of the technique. | Trial resin infiltration performed on the buccal surface of tooth **36** (NaOCl deproteinization, HCl etching, ethanol drying, Icon® (DMG) resin infiltration). | Immediate satisfactory esthetic improvement. Protocol validated before treating anterior teeth. |
| **Treatment session 2** | Mandibular anterior teeth. | Resin infiltration protocol performed (rubber dam isolation, 5% NaOCl deproteinization, one or two etching cycles according to lesion response, ethanol application, resin infiltration, light curing). | Immediate masking of enamel opacities with high patient satisfaction. |
| **Treatment session 3** | Maxillary anterior teeth. | Same infiltration protocol as previous session. | Significant improvement of smile esthetics. |
| **Treatment sessions 4–5** | Maxillary and mandibular premolars. | Resin infiltration performed on remaining teeth. | Completion of esthetic treatment with generalized masking of hypomature lesions. COHIP-SF improved to **62**. |
| **1-week follow-up** | Clinical assessment. | No additional treatment required. | Stable esthetic result and excellent patient satisfaction. |
| **6-month follow-up** | Orthodontic treatment initiated. | Conventional orthodontic bracket bonding on infiltrated enamel. Regular professional prophylaxis and topical fluoride varnish every 6 months. | No bracket debonding reported; infiltrated enamel remained clinically stable. |
| **3-year follow-up** | Completion of orthodontic treatment. | Clinical evaluation. | Stable esthetic appearance without discoloration, surface deterioration, polishing or retreatment. Patient remained highly satisfied with the treatment outcome. |

**Supplementary Table 1. Historical and current information from this episode of care organized as a timeline.**

| **Appointment** | **Teeth treated** | **Clinical procedures** |
| --- | --- | --- |
| Preliminary trial | Tooth 36 (mesial half of buccal surface) | Isolation, two etching cycles (2 min), ethanol application (60 s), resin infiltration (3 min + 1 min), light curing (40 s) |
| Session 1 | Mandibular anterior teeth (33–43) | Rubber dam isolation, 5% NaOCl deproteinization (60 s), 15% HCl etching (two cycles where required), ethanol drying (60 s), Icon® (DMG) resin infiltration (3 min + 1 min), glycerin application, final light curing |
| Session 2 | Maxillary anterior teeth (13–23) | Same protocol as Session 1; one or two etching cycles according to lesion response to ethanol |
| Session 3 | Mandibular premolars (34–35 and 44–45) | Standard resin infiltration protocol |
| Session 4 | Maxillary premolars (14–15 and 24–25) | Standard resin infiltration protocol |
| Follow-up visits | All treated teeth | Clinical examination, professional cleaning, fluoride varnish application during orthodontic treatment, assessment of esthetic stability |

**Supplementary Table 2. Summary of treated teeth and clinical procedures**
